# External validity of randomized clinical trials in vascular surgery: systematic review of demographic factors of patients recruited to randomized clinical trials with comparison to the National Vascular Registry

**DOI:** 10.1093/bjsopen/zrae156

**Published:** 2025-03-19

**Authors:** Joseph Cutteridge, Joseph Barsby, Samuel Hume, Hamish A L Lemmey, Regent Lee, Katarzyna D Bera

**Affiliations:** Specialised Foundation School, York and Scarborough Teaching Hospitals NHS Foundation Trust, York, UK; Department of Health Sciences, Faculty of Sciences, University of York, York, UK; Foundation School, Newcastle upon Tyne Hospitals NHS Foundation Trust, Newcastle, UK; Oxford Medical School, Oxford University, Oxford, UK; Oxford Medical School, Oxford University, Oxford, UK; Oxford Medical School, Oxford University, Oxford, UK; Nuffield Department of Surgical Sciences, University of Oxford, Oxford, UK; Vascular Surgery Department, John Radcliffe Hospital, Oxford University NHS Foundation Trust, Oxford, UK; Nuffield Department of Surgical Sciences, University of Oxford, Oxford, UK; Vascular Surgery Department, John Radcliffe Hospital, Oxford University NHS Foundation Trust, Oxford, UK

## Abstract

**Background:**

Evidence-based medicine relies on randomized clinical trials, which should represent the patients encountered in clinical practice. Characteristics of patients recruited to randomized clinical trials involving vascular index operations (carotid endarterectomy, abdominal aortic aneurysm repair, infrainguinal bypass and major lower limb amputations) were compared with those recorded in the National Vascular Registry across England and Wales.

**Methods:**

MEDLINE, Embase, Web of Science, CENTRAL, clinicaltrials.gov and World Health Organization International Trials Registry Platform (CRD42021247905) were searched for randomized clinical trials involving the index operations. Demographic (age, sex, ethnicity) and clinical (co-morbidities, medications, body mass index, smoking, alcohol, cognition) data were extracted, by operation. Characteristics of operated on patients were extracted from publicly available National Vascular Registry reports (2014–2020). All findings are reported according to PRISMA guidelines. Rayyan.AI, Excel and GraphPad Prism were used for screening and analysis.

**Results:**

A total of 307 randomized clinical trials (66 449 patients) were included and compared with National Vascular Registry data for 119 019 patients. Randomized clinical trial patients were younger across all operations; for carotid endarterectomy, bypass and major lower limb amputation randomized clinical trials, there were differences in female patient representation. Further comparisons were limited by the insufficient baseline data reporting across randomized clinical trials, though reporting improved over decades. National Vascular Registry reports lacked information on patient factors such as patient ethnicity or body mass index.

**Conclusions:**

There are significant differences in demographic and clinical factors between patients recruited to vascular surgery randomized clinical trials and the real-world National Vascular Registry vascular surgery patient population. Minimum reporting standards for baseline data should be defined to allow future randomized clinical trials to represent real-world patient populations and ensure the external validity of their results.

## Teaser

Practicing evidence-based medicine requires that the patients we encounter in daily practice are represented in the populations recruited to randomized clinical trials. In our work, we show that incompleteness of vascular surgery RCT reporting on patient data limits the applicability of their findings.

## Introduction

Evidence-based medicine is the foundation of modern clinical practice, with the best available evidence used to inform clinical decision-making and guideline development. Vascular surgery research has produced a high volume of randomized clinical trials (RCTs), followed by systematic reviews and meta-analyses, which have influenced the management of vascular disease internationally^1,2^.

All research needs to have high internal validity, to ensure that any observed differences between treatment groups can be attributed directly to the intervention being tested and establishing a causal relationship with the study environment. However, studies ought to be designed in a way that allows results to be generalizable to real-world settings and daily clinical practice. This involves recruiting study populations that accurately reflect the demographic and clinical characteristics of real-world patient populations. Therefore, while maintaining high internal validity is essential for the accuracy of study results, balancing this with high external validity is critical for the applicability and relevance of the research to modern clinical practice outside of research settings.

Basic demographic and clinical factors, such as age and co-morbidities, contribute to a patient’s suitability for surgery^3^. Furthermore, there is gathering evidence that previously overlooked characteristics such as sex^4^ and ethnicity^5^ also influence surgical outcomes. For example, a US study found that black men experience a higher postoperative mortality rate than other demographic groups^6^. Yet, research to date seems to have prioritized internal validity over external validity, meaning beneficial effects from trial settings do not always translate into real-world patient populations^7^. The previously reported beneficial effect of dual antiplatelet therapy for coronary stenting was greatly diminished when the study population was adjusted to reflect real-world patient characteristics^8^.

In the UK, vascular surgery activity is captured on the National Vascular Registry (NVR), providing an overview of four major operation types and details of the patient population^9^. The operations reported by the NVR are major lower limb amputations (MLLAs, both transtibial and transfemoral amputations), carotid endarterectomy (CEA, for symptomatic and asymptomatic carotid stenosis), endovascular and open repair of abdominal aortic aneurysms, and infrainguinal bypass surgery. The purpose of this research was to study the demographic and clinical factors of patients recruited to vascular surgery RCTs and compare against those reported by the NVR.

## Methods

### Systematic review

The protocol for the systematic review was prospectively registered with the International Prospective Register of Systematic Reviews (CRD42021247905). This review was conducted in agreement with the Preferred Reporting Items for Systematic Reviews and Meta-Analyses (PRISMA) reporting guidelines and followed the recommendations set by the Cochrane Collaboration^10,11^. The PRISMA checklist can be found in *Supplementary appendix 1*. RCTs that included one of the four major operations reported in the NVR were included irrespective of wider rationale or context of the RCTs, for example RCTs that studied types of anaesthesia were included. Studies that recruited and/or randomized patients after an operation had been completed were excluded, as were studies with fewer than 30 participants in total. Studies published in more than one report, such as substudies, nested studies or follow-up reports, were combined as they refer to the same patient population. Studies with outstanding results or minimal information available were included if, after contacting the investigators, a response with further information was received.

### Search strategy and data extraction

MEDLINE, Embase, CENTRAL, Web of Science and trial databases (clinicaltrials.gov and WHO International Clinical Trials Registry Platform) were searched from 1980 to 2021 for RCTs of vascular index operations. An updated complete search was conducted on 28 February 2023, and a further focused search of publications of the past year was undertaken in November 2023. A detailed search strategy is available in *Supplementary appendix 2*. Conference abstracts of major international conferences in the fields of vascular surgery were screened. Three reviewers independently screened the titles and abstracts identified by the literature search. Any disagreement was resolved by consensus after discussion or by arbitration by a fourth author. A data collection form was created and piloted on a subset by three authors (K.D.B., J.C. and J.B.); remaining data were extracted by authors (J.C., J.B., S.H., H.L.) independently. Disagreements were resolved through discussion or by arbitration by a further author (K.D.B.). Information was extracted regarding study characteristics (setting, sample size, intervention and comparator(s)) and patient demographic and clinical characteristics (age, sex, ethnicity, co-morbidities, medications, body mass index (BMI), cognition, smoking and alcohol intake). Data were extracted from the NVR annual reports, from 2015 to 2020 inclusively. Reports from subsequent years were not included due to the distorting effect of the COVID-19 pandemic on vascular surgery practice in the UK, with elective procedures cancelled, altering the demographic configuration of the population. For each of the four operations, all available patient characteristics were recorded. The NVR was approached to cross-check the extracted information as some reports spanned 2 years and some patients might have been represented in two reports.

### Data analysis

Studies were grouped by type of operation. The selection of patient variables for assessment (*Table 1*) was derived from data collected in the NVR and other key variables that are known to be risk factors for cardiovascular disease and/or operative outcomes. This was done because at present, there is no defined core reporting standard for baseline data within vascular surgery RCTs. Examples of additional variables include ethnicity^5^, BMI^12^, alcohol use^13,14^ and baseline cognition^15^. ASA data was also collected from RCTs and the NVR; however, due to inconsistent methods of reporting (ranges *versus* individual values), appropriate analysis could not be conducted. Where median and interquartile range were given, a web template^16^ was used for estimation of mean and standard deviation as described previously^17^. Means and standard deviations of the pooled cohort were calculated using the freely available online resource StatsToDo^18^. GraphPad PRISM 10.2.3 was used for statistical analysis and the creation of figures.

**Table 1:** Reporting of demographic and clinical characteristics from RCTs

Reported parameter	AAA repair (out of 77 RCTs)	CEA (out of 113 RCTs)	Bypass (out of 94 RCTs)	MLLA (out of 23 RCTs)
Number of patients (total: 66 449)	8724	27 395	27 591	2739
Age	76 (99)	109 (96)	87 (93)	19 (83)
Male	75 (97)	105 (93)	81 (86)	21 (91)
Ethnicity	3 (4)	6 (5)	16 (17)	2 (9)
Any co-morbidity	49 (64)	87 (77)	83 (88)	14 (61)
Diabetes mellitus	45 (58)	82 (73)	81 (86)	14 (61)
Hypertension	47 (62)	81 (72)	63 (67)	4 (17)
Any antiplatelet	11 (14)	21 (19)	14 (15)	1 (4)
Aspirin	11 (14)	21 (19)	14 (15)	1 (4)
Clopidogrel	4 (5)	11 (10)	9 (10)	1 (4)
Lipid-lowering agent	16 (21)	13 (12)	12 (13)	0 (0)
BMI	26 (34)	28 (25)	18 (1)	3 (13)
Smoking	42 (55)	61 (54)	61 (65)	5 (22)
Alcohol	1 (1)	2 (2)	4 (4)	1 (4)
Cognition	0 (0)	5 (4)	0 (0)	0 (0)

Values are *n* (%). RCT, randomized clinical trial; MLLA, major lower limb amputation; AAA, abdominal aortic aneurysm; CEA, carotid endarterectomy; BMI, body mass index.

For comparison between the RCT cohort and the NVR cohort, the unpaired one-tailed *t* test was used for age and Fisher’s exact test for sex. Where more than 5% of patient data was missing within an operative group, no comparison was attempted, as any such comparison would not be fair or valid, resulting in confidence intervals too wide to be of any real value^19^. Where comparison was not possible, plots of RCT data were generated to enable visualization.

## Results

### RCTs

**Fig. 1:**
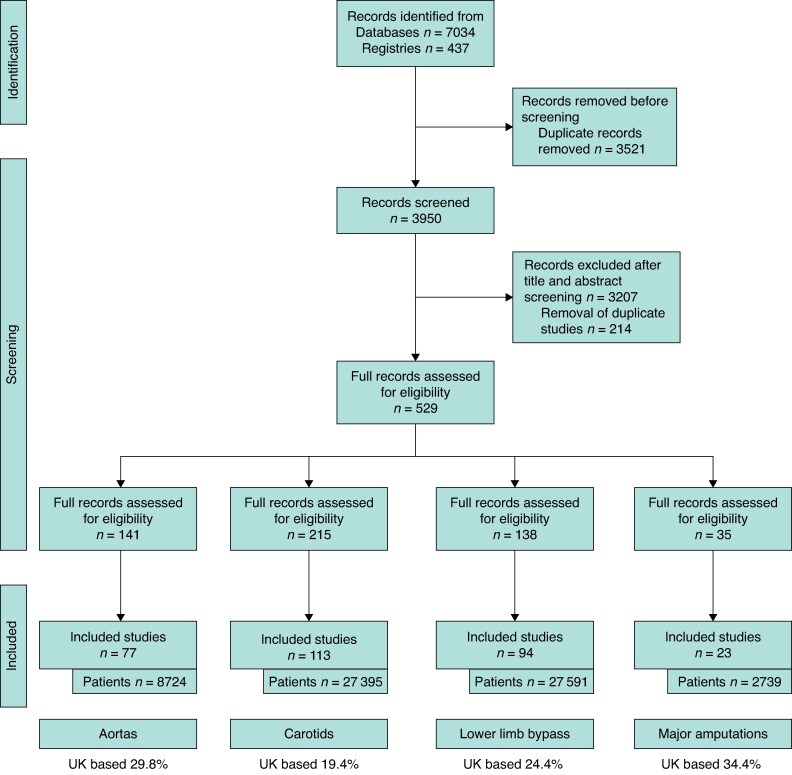
PRISMA flow diagram including record identification, screening and selection

**Fig. 2:**
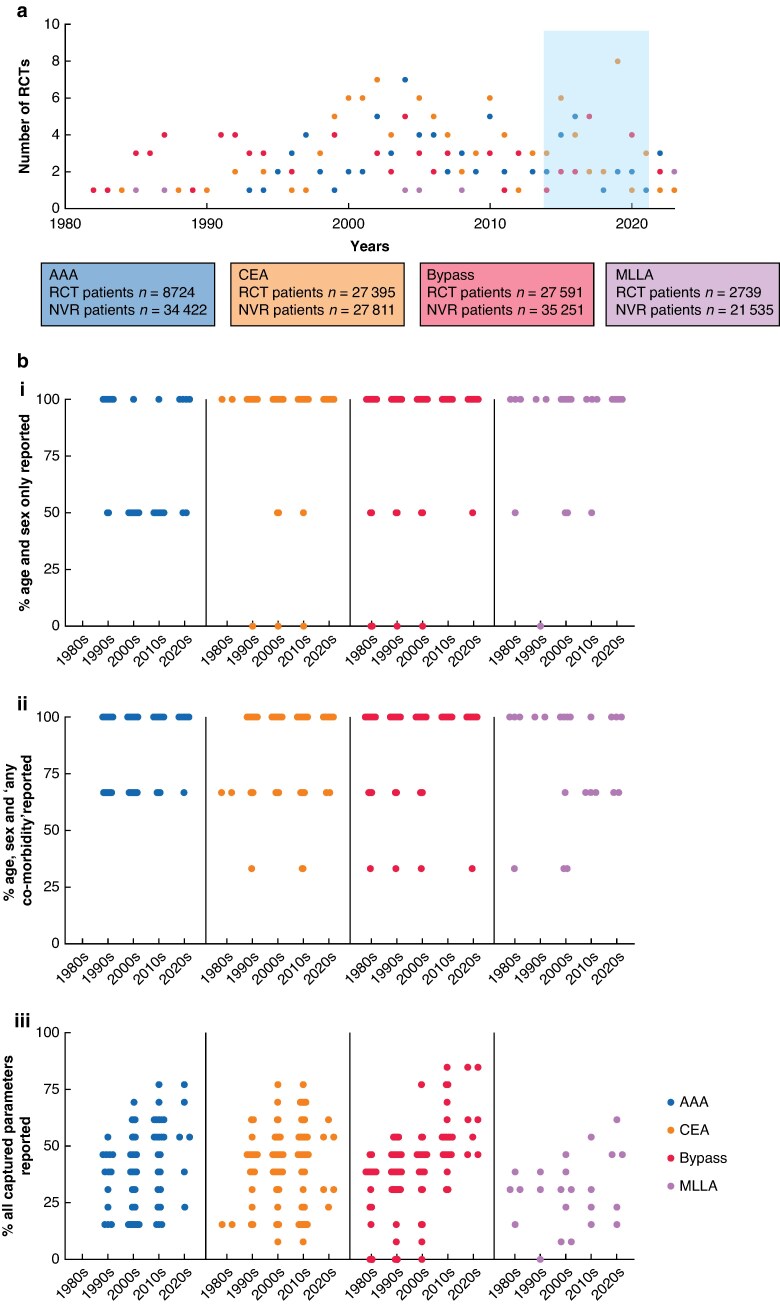
RCTs over the years

The search identified 7471 records, 3950 abstracts were screened and 529 full-text articles were assessed. A total of 307 RCTs were included in the final analysis (PRISMA, *Fig. 1*), of which 77 involved an abdominal aortic aneurysm (AAA) repair, 112 involved an CEA, 93 involved an infrainguinal bypass and 23 involved a MLLA. The included RCTs involved a total of 66 449 patients undergoing one of the mentioned vascular surgical operations as part of the trial (*Table 1*). Details of the included studies can be found in *Table S1*. Some 24% of studies included UK participants. The majority of included RCTs had fewer than 200 participants and RCTs were evenly distributed across the included decades (*Fig. 2a*). Some 74.7% of all aortic operations in the NVR were elective; 59.2% of all emergency or elective AAA repairs were endovascular rather than open.

**a** Publications of RCTs across years, with colour representing the type of operation. The blue area denotes the interval of extracted NVR reports. **b** Reporting of demographic and clinical factors by RCTs across decades. i: age and sex only. ii: age, sex and ‘any co-morbidity’. iii: all captured parameters. RCT, randomized clinical trial; NVR, National Vascular Registry; MLLA, major lower limb amputation; AAA, abdominal aortic aneurysm; CEA, carotid endarterectomy.

### NVR

Publicly available NVR reports included information on patient age, sex, presence of co-morbidities such as diabetes mellitus or hypertension, their smoking status and whether they were prescribed an antiplatelet or lipid-lowering agent. The total number of patients included in the NVR was 119 019.

### Reporting of baseline data from RCTs

Analysis of RCTs showed discrepancies in reporting of patient characteristics across the four operation types (*Table 1*). Patient age was reported almost universally, with patient sex reported to a lesser degree. Reporting of age and sex increased over the decades and all studies published after 2010 report on patients’ age and sex (*Fig. 2b*). The reporting of ‘any co-morbidity’ also increased, albeit to a lesser degree. The reporting of other demographic and clinical factors did not follow this trend and remains insufficient even through to the 2020s. However, reporting of all included parameters showed significant improvement over time for RCTs of AAA (r_S_ = 0.31, 95% c.i 0.08 to 0.50), Spearman’s rank correlation test) and infrainguinal bypass (r_S_ = 0.58, 95% c.i. 0.43 to 0.71, Spearman’s rank correlation test).

### Comparison of RCT and NVR populations

**Fig. 3:**
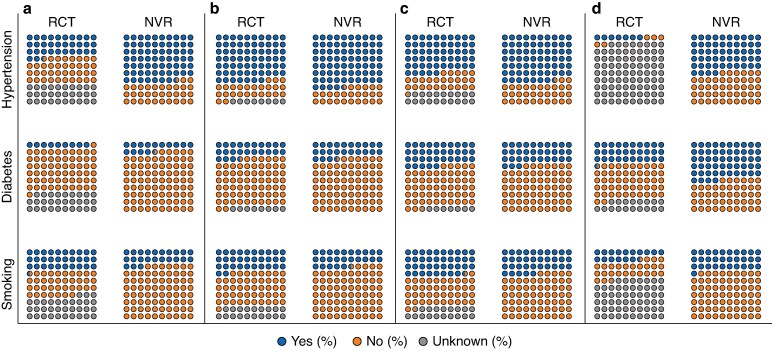
**Visual representation of the percentage for patients with hypertension, diabetes, as well as current/former smokers by type of operation**.

Across all included operations, the age of patients recruited to RCTs was younger than patients in the registry cohort (*Table 2*). There was also a significant difference in the male to female ratio between the trial and registry cohorts. Of note, more female patients were included in the infrainguinal bypass and MLLA RCT cohorts and fewer in the CEA cohort—although the clinical relevance of this might be low. Unfortunately, all captured parameters, other than age and sex, were reported with more than 5% of patient-level data missing within each operative group, preventing statistical analysis. *Figure 3* shows an overview of the NVR and RCT cohorts, displaying the percentage of patients who have a diagnosis of hypertension, diabetes mellitus, and those who are current or former smokers.

Comparison of randomized clinical trial (RCT) cohort (left) with National Vascular Registry (NVR) cohort (right), by type of operation: a = abdominal aortic aneurysm, b = carotid endarterectomy, c = infrainguinal bypass, d = major lower limb amputation. Grey dots represent the percentage of patients recruited to RCTs where no information was provided and therefore their status is unknown.

**Table 2:** Comparison of RCT and NVR data for age and sex information, by type of operation

Operation	RCT	NVR	*P*
**Age (years), mean(s.d.)**			
AAA	70.8(7.8) (N = 8724)	74.8(8.3) (N = 34 442)	<0.0001
CEA	68.7(8.7) (N = 27 395)	72.0(10.1) (N = 27 811)	<0.0001
Bypass	67.6(12.1) (N = 27 591)	68.7(11.4) (N = 35 251)	<0.0001
MLLA	64.8(16.3) (N = 2739)	68.4(13.0) (N = 21 535)	<0.0001
**Sex (N female/N total reported population)**			
AAA	942/7861 (12.0%)	4268/34 422 (12.4%)	0.160
CEA	8227/27 395 (30.7%)	9094/27 811 (32.7%)	<0.0001
Bypass	6867/25 372 (27.1%)	9377/35 251 (26.6%)	<0.0001
MLLA	990/2654 (37.3%)	6245/21 535 (29.0%)	<0.0001

For the RCT population this refers to the total N of patients with reported information. Statistical test used was unpaired one-tailed *t* test (age) and Fisher's exact test (sex). RCT, randomized clinical trial; NVR, National Vascular Registry; MLLA, major lower limb amputation; AAA, abdominal aortic aneurysm; CEA, carotid endarterectomy.

## Discussion

This systematic review and analysis has demonstrated that the demographic characteristics of patients recruited to vascular surgery RCTs varies considerably compared with the NVR population. The planned comparison of the RCT and registry population across all demographic factors was, however, limited by the insufficient reporting of patient factors across many of the RCTs.

The strength of this review and comparison is the strict methodological process. Cochrane Collaboration and PRISMA recommendations were followed. A comprehensive search was conducted encompassing multiple databases and clinical trial registries to ensure all relevant studies were captured. All screening and data extraction were done in duplicate. To the authors’ knowledge, this systematic review is the first to examine the demographic and clinical differences across all major vascular operations, with a total research population of 66 449 patients and registry population of 119 019, offering the most comprehensive overview of such discrepancies to date.

This work has limitations. The registry population consisted of only patients in England and Wales; however, between 19.4% and 34.4% of the included studies were entirely or partially based in the UK. The USA was the largest contributor of RCTs to this review, which has a distinct population when compared with the UK, with marked differences in terms of demographic and clinical factors. This includes factors such as ethnicity (white population of the USA is 61.6%, in the UK it is 81.7%)^20,21^, prevalence of obesity (41.9% in the USA *versus* 25.9% in the UK)^22,23^ and diabetes (10% in the USA *versus* 6.42% in the UK)^24,25^. However, the analysis was mostly limited due to the underreporting of factors in the RCT population—therefore, comparison to other registry populations would have been affected in the same way. The NVR is a surgeon self-reported registry, therefore, it remains at risk of reporting bias—though it would likely mean that more co-morbid and/or complex patients are underreported, which would likely further widen the divide to the trial population. The current comparison spans four decades of vascular surgery RCTs, and it is acknowledged that attitudes to research participation and recruitment might have changed over time: recruitment to acute stroke trials slowed or decreased from the 1990s to 2014 and there is a recognized burden to patients participating in research, which might have further increased with changes to RCT data collection and follow-up over the years^26,27^.

Discrepancies between clinical and research vascular surgery populations have been reported elsewhere using different approaches. A German study comparing the characteristics of patients undergoing carotid stenting in RCTs to a sample of a real-world clinical population revealed significant differences in age, co-morbidities and medications^28^. In 2009, Hoel *et al*. demonstrated an underrepresentation of women and minority ethnic groups in US-based RCTs compared with the Nationwide Inpatient Sample Database^29^; the discrepancy in the outcomes of these groups was discussed in 2010^30^. The present work complements this existing body of research, providing a systematic review of all RCTs involving vascular surgical operations, comparing it to the national registry of patients.

Age remains a key predictor of postoperative outcomes^31,32^, so the improved reporting over the decades is important, especially as today’s patients live longer than those in the early vascular studies^33^. It is crucial to recruit patients to RCTs that represent the age of our patient population. Currently, we are not meeting this essential requirement. A significant difference in the sex ratio of RCT and clinical populations is also reported here, although surprisingly the proportion of recruited women did not always point in the same direction. Some of these results might be explained by the trends in vascular surgical operations seen over time and the comparison of a wider RCT timeframe to a shorter NVR interval. In the UK, the percentage of women undergoing CEA increased from 2011 to 2017^34^, and the rate of women requiring an MLLA reduced between 2006 and 2018^35^; both open and endovascular revascularization in women is also reported to have reduced between 2000 and 2019. The changing trends in real-world operations might explain the observed over- and underrepresentation of women in those operations when compared with the RCT population^36^. Importantly, even if female patient representation reflects the real-world patient populations, this subgroup might have different outcomes. The underrepresentation of women in some AAA trials has left uncertainty with regards to threshold AAA diameter to treat or best modality of treatment^37–40^—the upcoming women's aneurysm research: repair immediately or routine surveillance (WARRIORS) trial is designed to answer some of the questions^41^, decades after the landmark AAA trials concluded. Marcaccio *et al*. highlighted this underrepresentation of female patients in aortic trials, particularly EVAR trials, within the food and drug administration’s (FDA) medical devices database, advocating the need for minimum thresholds for female participation^42^.

Differences were also found in the co-morbidities of these two cohorts: the prevalence of hypertension appears lower in AAA RCTs *versus* the NVR, as is the prevalence of diabetes in MLLA RCTs *versus* the NVR (*Fig. 3*). However, inadequate reporting limits the ability to conduct statistical comparisons. Hypertension is a key co-morbidity that leads to the development of atherosclerosis and its complications, including ischaemic heart disease (IHD), stroke and chronic renal failure, which are known to affect perioperative morbidity and mortality rates across all index surgeries^43,44^. Similarly, diabetes is a significant contributor to postoperative complications, especially after lower limb surgery, and can reduce the effectiveness of additional medical treatments such as antiplatelet agents^45–47^. Therefore, trial populations with reduced numbers of hypertensive and diabetic patients might be less likely to report certain postoperative complications, compromising their external validity.

Extreme BMI is also associated with poor cardiovascular outcomes, with a BMI of <20 kg/m^2^ and >35 kg/m^2^ strong markers of adverse prognosis^12^. Yet, BMI was only reported in 77 of 307 publications, resulting in readers being unable to judge if BMI was equally distributed across treatment groups, and to check that the trial population reflects that of their population of interest. Baseline cognition was poorly reported across RCTs, despite its significant prevalence among the vascular surgery population, estimated at 61%, and its strong association to postoperative delirium^15^. Although one article outside the included set, an RCT evaluating the effect of comprehensive geriatric assessment, did measure cognitive performance, it did not provide a breakdown of patient demographics by type of operation, rendering it unsuitable to include in the analysis.

Despite the poor reporting of many parameters across RCTs, it is important to recognize that ethnicity, BMI, alcohol consumption, and cognitive baseline are not routinely reported by the NVR, preventing any comparison to the UK vascular population.

RCTs remain the accepted gold standard of evidence that influences clinical decision-making, shapes clinical guidelines and ultimately patient care. However, if trials fail to recruit representative populations, the promising results seen within the study population might be attenuated when translated across to real-world populations^48^. Conversely, poor external validity may underestimate the real-world effect of novel treatments. A French research group found that 30.1% of hospitalized patients with peripheral arterial disease (PAD) would have been eligible for the cardiovascular outcomes for people using anticoagulation strategies (COMPASS)^49^ or vascular outcomes study of ASA along with rivaroxaban in endovascular or surgical limb revascularization for peripheral artery disease (VOYAGER-PAD)^50^ trials, which investigated a regime of rivaroxaban 2.5 mg plus aspirin^51^. Yet, within this eligible subset, major adverse events were significantly higher than those seen in the control arms for both studies^51^, thus the COMPASS and VOYAGER-PAD medication regime may have greater benefits in the real-world population.

This systematic review and analysis raises the need for minimum core reporting standards for baseline data within vascular surgery RCTs. CONSORT added the requirement to report baseline data and clinical characteristics in its 2001 revision. The latest CONSORT guidance provides clear reporting standards regarding outcomes, with clearly defined primary and secondary outcomes^52^; however, with regards to the study population, current guidance is still very limited, stating, ‘Baseline data: a table showing baseline demographic and clinical characteristics for each group’. The Core Outcomes Measures in Effectiveness Trials (COMET) initiative^53^ provides more in-depth guidance via links to previously published reporting standards for different conditions, including PAD^54^ and carotid atherosclerosis^55^; however, it does not include standards for AAA or amputation surgery. Additionally, the focus of these studies is outcome data, with considerable variability in the range and depth of suggestions regarding baseline data. The authors of this review advocate for a core reporting standard for baseline data within vascular surgery RCTs. The inclusion of all parameters extracted for this review as well as specific ASA grade of patients (not ranges) might form the basis for a Delphi consensus to define a comprehensive overview of demographic and clinical characteristics for trial populations. The notion of a condition-specific core reporting standard, or core descriptors, has also been recently advocated by others^56^, reflecting a growing, widespread desire to improve clinical characterization, reproducibility and external validity.

Key factors contributing to the discrepancies between trial and real-world populations include differences between explanatory and pragmatic trial designs, the former of which often selects younger, healthier participants. Recruitment settings, often biased toward academic centres, can further skew participant characteristics. Over the last decades, the delivery of clinical research has changed: trials and their outcomes are encouraged to be prospectively registered^57^, it is more common practice to publish protocols to allow scrutiny by peers, and the value of feasibility or pilot RCTs is well recognized with recommendations for standardization^58^. Studies such as those focusing on (mostly male) veterans^40^ have become less common and there has been a welcome rise in more pragmatic trials even in areas previously considered less suitable, such as emergency surgery^59^ and prehospital interventions^60^.

There are other trends within clinical research that may help improve the external validity of future trials. The rise of adaptive platform trials, including the landmark randomized evaluation of COVID-19 therapy (RECOVERY) trial, have demonstrated that research can be expertly integrated into clinical practice to deliver timely results. At the height of the pandemic RECOVERY trial participants comprised 10–12% of all patients in UK hospitals and this has been estimated to have saved over 1 million lives^48^. The scale of the trial inevitably results in the spectrum of patients recruited reflecting that of the target population, producing results with strong external validity. The rapid advancement of novel technologies and techniques, including artificial intelligence (AI) and machine learning (ML), may soon produce models that can effectively assess the risks and benefits of vascular surgery for individual patients. Importantly, AI research is data driven as opposed to classical hypothesis-driven research, making data integrity more important than ever. If these models are built on data that do not reflect the target population, they will incorporate the selection bias of the literature, leading to inaccurate predictions.

Whilst no one questions the importance of RCTs in evidence based medicine (EBM), the need to recruit from real-world populations needs to emphasized. More inclusive recruitment strategies and standardized reporting of baseline characteristics, such as ethnicity and co-morbidities, would improve the external validity of future vascular surgery trials. In addition, clinical guidelines benefit from interpreting RCT outcomes in the context of and alongside well designed cohort and registry studies. This will provide significantly enhanced insights to clinicians and authors of clinical guidelines, advancing care for the populations they serve.

## Supplementary Material

zrae156_Supplementary_Data

## Data Availability

The data supporting the findings are available within the article and its supplementary information. Raw data that support the findings of this work can be made available from the corresponding author, upon reasonable request.
