# Supplementary material for: External validity of randomized clinical trials in vascular surgery: systematic review of demographic factors of patients recruited to randomized clinical trials with comparison to the National Vascular Registry
Source: BJS Open. 2025 Mar 19;9(2):zrae156. doi: 10.1093/bjsopen/zrae156 (PMC11921775; doi:10.1093/bjsopen/zrae156)
Supplement: zrae156_Supplementary_Data [file zrae156_supplementary_data.zip › S2Search strategy.docx]

**Supplementary Search Strategy**

Preliminary, exploratory searches were conducted on all databases and registries prior to initial, formal searches. The primary goal was a broad search strategy that would capture all desired randomised controlled trials between 1981 and 2023, yet pragmatically focussed to allow reviewers to screen titles. Where possible, limits were used to focus searches. Limits included publication dates (all between 1981 and 2023) and study type (limiting to RCTs only). MeSH terms were used where available. Searches were conducted initially between July and August 2021, and were repeated on 28/02/2023. Repeated searches were identical other than limited by date. Details of limits and title numbers from initial and repeated searches are outlined below.

# **Pubmed**

| **#** | **Query** | **Filters** | **Search Details** | **Results** |
| --- | --- | --- | --- | --- |
| 1 | ((infrainguinal OR infra-inguinal OR iliac-femoral OR iliofemoral OR aortic-femoral OR aortofemoral OR femorofemoral OR femoro-femoral OR fem-fem OR femoropopliteal OR femoro-popliteal OR fem-pop OR femorotibial OR femoro-tibial OR fem-tib OR femorodistal OR femoro-distal OR lower extremity OR lower limb) AND bypass) | Randomized Controlled Trial, from 1981 - 2021 | (("infrainguinal"[All Fields] OR "infra-inguinal"[All Fields] OR "iliac-femoral"[All Fields] OR "iliofemoral"[All Fields] OR "aortic-femoral"[All Fields] OR "aortofemoral"[All Fields] OR "femorofemoral"[All Fields] OR "femoro-femoral"[All Fields] OR "fem-fem"[All Fields] OR "femoropopliteal"[All Fields] OR "femoro-popliteal"[All Fields] OR "fem-pop"[All Fields] OR "femorotibial"[All Fields] OR "femoro-tibial"[All Fields] OR "fem-tib"[All Fields] OR "femorodistal"[All Fields] OR "femoro-distal"[All Fields] OR ("lower extremity"[MeSH Terms] OR ("lower"[All Fields] AND "extremity"[All Fields]) OR "lower extremity"[All Fields]) OR ("lower extremity"[MeSH Terms] OR ("lower"[All Fields] AND "extremity"[All Fields]) OR "lower extremity"[All Fields] OR ("lower"[All Fields] AND "limb"[All Fields]) OR "lower limb"[All Fields])) AND ("bypass"[All Fields] OR "bypassed"[All Fields] OR "bypasses"[All Fields] OR "bypassing"[All Fields])) AND ((randomizedcontrolledtrial[Filter]) AND (1981:2021[pdat])) | 356 |
| 2 | "Amputation"[Mesh] OR "Amputation, Traumatic"[Mesh] OR ((Boyd OR Chopart OR Symes OR below knee OR below the knee OR below-the-knee OR lower limb OR partial limb OR transtibial OR trans-tibial OR transfemoral OR trans-femoral) AND Amputation) | Randomized Controlled Trial, from 1981 - 2021 | ("Amputation"[MeSH Terms] OR "amputation, traumatic"[MeSH Terms] OR (("Boyd"[All Fields] OR ("chopart"[All Fields] OR "chopart s"[All Fields]) OR ("syme s"[All Fields] OR "symes"[All Fields]) OR ("below"[All Fields] AND ("knee"[MeSH Terms] OR "knee"[All Fields] OR "knee joint"[MeSH Terms] OR ("knee"[All Fields] AND "joint"[All Fields]) OR "knee joint"[All Fields])) OR ("below"[All Fields] AND ("knee"[Journal] OR ("the"[All Fields] AND "knee"[All Fields]) OR "the knee"[All Fields])) OR "below-the-knee"[All Fields] OR ("lower extremity"[MeSH Terms] OR ("lower"[All Fields] AND "extremity"[All Fields]) OR "lower extremity"[All Fields] OR ("lower"[All Fields] AND "limb"[All Fields]) OR "lower limb"[All Fields]) OR (("partial"[All Fields] OR "partials"[All Fields]) AND ("extremities"[MeSH Terms] OR "extremities"[All Fields] OR "limb"[All Fields])) OR "transtibial"[All Fields] OR "trans-tibial"[All Fields] OR ("transfemoral"[All Fields] OR "transfemorally"[All Fields]) OR "trans-femoral"[All Fields]) AND ("amputate"[All Fields] OR "amputated"[All Fields] OR "amputating"[All Fields] OR "Amputation"[MeSH Terms] OR "Amputation"[All Fields] OR "amputations"[All Fields] OR "amputed"[All Fields]))) AND ((randomizedcontrolledtrial[Filter]) AND (1981:2021[pdat])) | 624 |
| 3 | "Aortic Aneurysm, Abdominal"[Mesh] | Randomized Controlled Trial, from 1981 - 2021 | ("aortic aneurysm, abdominal"[MeSH Terms]) AND ((randomizedcontrolledtrial[Filter]) AND (1981:2021[pdat])) | 436 |
| 4 | "Endarterectomy, Carotid"[Mesh] | Randomized Controlled Trial, from 1981 - 2021 | ("endarterectomy, carotid"[MeSH Terms]) AND ((randomizedcontrolledtrial[Filter]) AND (1981:2021[pdat])) | 423 |

Identical searches were carried out with the above listed search details on the 28/02/2023, with the added limit of years (2021-2023). Search results were as listed below:

- Infrainguinal bypass: 22 titles
- Major lower limb amputation: 67 titles
- Abdominal aortic aneurysm: 13 titles
- Carotid endarterectomy: 11 titles

# **Medline**

Medline (Ovid MEDLINE® Epub Ahead of Print, In-Process & Other Non-Indexed Citations, Ovid MEDLINE® Daily and Ovid MEDLINE®) 1946 to present

**AAA**

1 exp Aortic Aneurysm, Abdominal/ 20205

2 limit 1 to yr="1981 -Current" 20008

3 limit 2 to randomized controlled trial **436**

**CEA**

1 exp Endarterectomy, Carotid/ 9106

2 limit 1 to yr="1981 -Current" 9093

3 limit 2 to randomized controlled trial **422**

**Amputation**

1 exp Amputation/ or exp Amputation, Traumatic/ 26952

2 ((Boyd or Chopart or Symes or below knee or below the knee or below-the-knee or lower limb or partial limb or transtibial or trans-tibial or transfemoral or trans-femoral) and Amputation).mp. [mp=title, abstract, original title, name of substance word, subject heading word, floating sub-heading word, keyword heading word, organism supplementary concept word, protocol supplementary concept word, rare disease supplementary concept word, unique identifier, synonyms] 7736

3 1 or 2 30371

4 limit 3 to yr="1981 - 2021" 23790

5 limit 4 to randomized controlled trial **492**

**Infrainguinal bypass**

1 ((infrainguinal or infra-inguinal or iliac-femoral or iliofemoral or aortic-femoral or aortofemoral or femorofemoral or femoro-femoral or fem-fem or femoropopliteal or femoro-popliteal or fem-pop or femorotibial or femoro-tibial or fem-tib or femorodistal or femoro-distal or lower extremity or lower limb) and bypass).mp. [mp=title, abstract, original title, name of substance word, subject heading word, floating sub-heading word, keyword heading word, organism supplementary concept word, protocol supplementary concept word, rare disease supplementary concept word, unique identifier, synonyms] 6954

2 limit 1 to yr="1981 -Current" 6569

3 limit 2 to randomized controlled trial **256**

Identical searches were conducted on 28/02/2023 with the following limitation:

2 limit 1 to yr="2021 -Current"

Titles yielded from each search are listed below:

- Abdominal aortic aneurysm: 13
- Carotid endarterectomy: 11
- Major amputation: 55
- Infrainguinal bypass: 15

# **Embase**

**AAA**

1 exp abdominal aortic aneurysm/ 7438

2 limit 1 to yr="1981 -Current" 7438

3 limit 2 to randomized controlled trial **137**

**Carotid endarterectomy**

1 exp Endarterectomy, Carotid/ 18968

2 limit 1 to yr="1981 -Current" 18734

3 limit 2 to randomized controlled trial **739**

**Amputation**

1 exp Amputation/ 49200

2 ((Boyd or Chopart or Symes or below knee or below the knee or below-the-knee or lower limb or partial limb or transtibial or trans-tibial or transfemoral or trans-femoral) and Amputation).mp. [mp=title, abstract, heading word, drug trade name, original title, device manufacturer, drug manufacturer, device trade name, keyword, floating subheading word, candidate term word] 12687

3 1 or 2 51038

4 limit 3 to yr="1981 -Current" 48671

5 limit 4 to randomized controlled trial **1055**

**Infrainguinal Bypass**

1 ((infrainguinal or infra-inguinal or iliac-femoral or iliofemoral or aortic-femoral or aortofemoral or femorofemoral or femoro-femoral or fem-fem or femoropopliteal or femoro-popliteal or fem-pop or femorotibial or femoro-tibial or fem-tib or femorodistal or femoro-distal or lower extremity or lower limb) and bypass).mp. [mp=title, abstract, heading word, drug trade name, original title, device manufacturer, drug manufacturer, device trade name, keyword, floating subheading word, candidate term word] 11727

2 limit 1 to yr="1981 -Current" 11164

3 limit 2 to randomized controlled trial **315**

Identical searches were conducted on 28/02/2023 with the following limitation:

2 limit 1 to yr="2021 -Current"

Titles yielded from each search are listed below:

- Abdominal aortic aneurysm: 70
- Carotid Endarterectomy: 55
- Major lower limb amputation: 267
- Infrainguinal bypass: 34

# **Web of Science**

**AAA**

(ALL=(Abdominal Aortic Aneurysm OR AAA OR TRIPLE*A) AND (TI=(RANDOMI*ED CONTROL TRIAL) OR TI=(RCT) OR TI=(RANDOMI*ED CONTROL TRIAL))) AND PY=(1981-2021)

**151 results**

**Carotid Endarterectomy**

ALL=(Carotid Endarterectomy) AND TI=(RANDOMI*ED CONTROL TRIAL OR RCT OR RANDOMI*ED CONTROL TRIAL) AND PY=(1981-2021)

**126 results**

**Amputation**

ALL=(Amputation OR ((Boyd or chopart or symes or below knee or below the knee or below-the-knee or lower limb or partial limb or transtibial or trans-tibial or transfemoral or trans-femoral) and Amputation)) AND TI=(RANDOMI*ED CONTROL TRIAL OR RCT OR RANDOMI*ED CONTROL TRIAL) AND PY=(1981-2021)

**247 Results**

**Infrainguinal Bypass**

ALL=((infrainguinal or infra-inguinal or iliac-femoral or iliofemoral or aortic-femoral or aortofemoral or femorofemoral or femoro-femoral or fem-fem or femoropopliteal or femoro-popliteal or fem-pop or femorotibial or femoro-tibial or fem-tib or femorodistal or femoro-distal or lower extremity or lower limb) and bypass) AND TI=(RANDOMI*ED CONTROL TRIAL OR RCT OR RANDOMI*ED CONTROL TRIAL) AND PY=(1981-2021)

**56 results**

Additional, identical searches were conducted on 28/02/2023 with an added limitation:

“AND PY=(2021-2023)”

Titles yielded from each search are listed below:

- Abdominal aortic aneurysm: 33
- Carotid endarterectomy: 10
- Major lower limb amputation: 81
- Infrainguinal bypass: 6

# **Cochrane Central Register of Controlled Trials (CENTRAL)**

**AAA**

#1 MeSH descriptor: [Aortic Aneurysm, Abdominal] explode all trees 594

#2 #1 AND ("RANDOMI*ED CONTROL TRIAL" OR "RANDOMI*ED CONTROLLED TRIAL" OR "RCT") with Publication Year from 2021 to 2023, with Cochrane Library publication date Between Jan 1981 and Jul 2021, in Trials (Word variations have been searched) 13

**Carotid Endarterectomy**

#1 MeSH descriptor: [Endarterectomy, Carotid] explode all trees 473

#2 #2 AND ("RANDOMI*ED CONTROL TRIAL" OR "RANDOMI*ED CONTROLLED TRIAL" OR "RCT") with Publication Year from 1981 to 2021, with Cochrane Library publication date Between Jan 1981 and Jul 2021, in Trials (Word variations have been searched) 2

**Amputation**

#1 Amputation OR ((Boyd or chopart or symes or below knee or below the knee or below-the-knee or lower limb or partial limb or transtibial or trans-tibial or transfemoral or trans-femoral) and Amputation) 3112

#2 #1 AND ("RANDOMI*ED CONTROL TRIAL" OR "RANDOMI*ED CONTROLLED TRIAL" OR "RCT") with Publication Year from 1981 to 2021, with Cochrane Library publication date Between Jan 1981 and Jul 2021, in Trials (Word variations have been searched) 85

**Infrainguinal bypass**

#1 ((((infrainguinal or infra-inguinal or iliac-femoral or iliofemoral or aortic-femoral or aortofemoral or femorofemoral or femoro-femoral or fem-fem or femoropopliteal or femoro-popliteal or fem-pop or femorotibial or femoro-tibial or fem-tib or femorodistal or femoro-distal or lower extremity or lower limb) and bypass))) 834

#2 #1 AND ("RANDOMI*ED CONTROL TRIAL" OR "RANDOMI*ED CONTROLLED TRIAL" OR "RCT") with Publication Year from 1981 to 2021, with Cochrane Library publication date Between Jan 1981 and Jul 2021, in Trials (Word variations have been searched) 18

Identical searches were undertaken again on 28/02/2023 with added limitation of with “Publication Year from 2021 to 2023” to line #2. Titles yielded from each search are listed below:

- Abdominal aortic aneurysm: 2
- Carotid endarterectomy: 1
- Major lower limb amputation: 20
- Infrainguinal bypass: 4

# **ClinicalTrials.gov**

**AAA**

condition or disease: Abdominal Aortic Aneurysm

study type: All

study results: All

results first posted: 01/01/1981 - 07/08/2021 (MM/DD/YYYY)

**titles: 31**

**Carotid endarterectomy**

condition or disease: carotid endarterectomy

study type: All

study results: All

results first posted: 01/01/1981 - 07/08/2021 (MM/DD/YYYY)

**titles: 19**

**Amputation**

condition or disease: Amputation OR ((Boyd or chopart or symes or below knee or below the knee or below-the-knee or lower limb or partial limb or transtibial or trans-tibial or transfemoral or trans-femoral) and Amputation)

study type: All

study results: All

results first posted: 01/01/1981 - 07/08/2021 (MM/DD/YYYY)

**titles: 46**

**Infrainguinal bypass**

9 searches each ran independently due to character limit in search box.

See below for ‘condition or disease’, numbered 1-9

Condition or disease:

1. (Femorofemoral OR femoro-femoral OR femoro femoral OR fem-fem OR femfem OR fem fem) AND bypass – **4**
2. (Femoropopliteal OR femoro-popliteal OR femoro popliteal OR fem-pop OR fempop OR fem pop) AND bypass – **23**
3. (Femoro-tibial OR femorotibial OR femoro tibial OR Fem-tib OR fem tib OR femib) AND bypass – **1**
4. (Infrainguinal OR infra-inguinal OR infra inguinal) AND bypass – **5**
5. (Iliofemoral OR ilio-femoral OR ilio femoral) AND bypass – **2**
6. Femoro-distal bypass – **0**
7. Lower extremity bypass – **30**
8. (Aortofemoral OR aorto-femoral OR aorto femoral OR aorto-fem OR aorto fem OR aortofem) AND bypass – **1**
9. Lower limb bypass – **30**

study type: All

study results: All

results first posted: 01/01/1981 - 07/08/2021 (MM/DD/YYYY)

Identical searches were conducted on 28/02/2023 with the following, amended limitation:

results first posted: 07/08/2021 – 02/28/2023 (MM/DD/YYYY)

Number of results yielded from each repeat search were as below:

- Abdominal aortic aneurysm: 13
- Carotid endarterectomy: 1
- Major lower limb amputation: 6
- Infrainguinal bypass: total 5
  - (7) Lower extremity bypass: 2
  - (9) Lower limb bypass: 3

# **WHO registry**

**AAA**

Title: ‘Abdominal aortic aneurysm’, synonyms included (abdominal; abdominal || aortic; aortic || aneurrysm; aneurysm), recruitment status: ALL, phases: ALL, registration dates: 01/01/1981-07/06/2021, with results - **13 records for 12 trials**

**Carotid Endarterectomy**

Title: ‘Carotid Endarterectomy’, synonyms included (carotid; carotid || endarterectomy; Endarterectomies; endarterectomy; Thromboendarterectomies), recruitment status: ALL, 01/01/1981-07/06/2021, with results - **18 records for 18 trials**

**Amputation**

Title: ‘Amputation OR ((Boyd or chopart or symes or below knee or below the knee or below-the-knee or lower limb or partial limb or transtibial or trans-tibial or transfemoral or trans-femoral) and Amputation)’, recruitment status: ALL, phases: ALL, registration dates: 01/01/1981-07/06/2021, with results – **44 records for 42 trials**

**Infrainguinal Bypass**

((infrainguinal or infra-inguinal or iliac-femoral or iliofemoral or aortic-femoral or aortofemoral or femorofemoral or femoro-femoral or fem-fem or femoropopliteal or femoro-popliteal or fem-pop or femorotibial or femoro-tibial or fem-tib or femorodistal or femoro-distal or lower extremity or lower limb) and bypass), with results – **4 records for 3 trials**

Identical searches were conducted on 28/02/2023, with the added limitation of: “registration dates: 07/06/2021-28/02/2023”

Titles yielded for each search are as below:

- **Abdominal aortic aneurysm: 0**
- **Carotid endarterectomy: 0**
- **Major lower limb amputation: 0**
- **Infrainguinal bypass: 0**
