# Supplementary material for: External validity of randomized clinical trials in vascular surgery: systematic review of demographic factors of patients recruited to randomized clinical trials with comparison to the National Vascular Registry
Source: BJS Open. 2025 Mar 19;9(2):zrae156. doi: 10.1093/bjsopen/zrae156 (PMC11921775; doi:10.1093/bjsopen/zrae156)
Supplement: zrae156_Supplementary_Data [file zrae156_supplementary_data.zip › S3List of all RCTs. docx.docx]

| **Aorta** | | | | | | |
| --- | --- | --- | --- | --- | --- | --- |
| **Study Identifier** | **Authors** | **Year of publication** | **Title** | **Journal** | **Number of trial participants** |  |
| **AO1** | Lunen, T. B. and Johansson, P. I. and Jensen, L. P. and Homburg, K. M. and Roeder, O. C. and Lonn, L. and Secher, N. H. and Helgstrand, U. and Carstensen, M. and Jensen, K. B. and Lange, T. and Sillesen, H. and Swiatek, F. and Nielsen, H. B. | 2018 | Administration of platelets to ruptured abdominal aortic aneurysm patients before open surgery: a prospective, single-blinded, randomised study | Transfus Med | 122 |  |
| **AO2** | Spark, J. I. and Chetter, I. C. and Kester, R. C. and Scott, D. J. | 1997 | Allogeneic versus autologous blood during abdominal aortic aneurysm surgery | Eur J Vasc Endovasc Surg | 50 |  |
| **AO3** | Leijdekkers, V. J. and Vahl, A. C. and Mackaay, A. J. and Huijgens, P. C. and Rauwerda, J. A. | 2006 | Aprotinin does not diminish blood loss in elective operations for infrarenal abdominal aneurysms: a randomized double-blind controlled trial | Ann Vasc Surg | 35 |  |
| **AO4** | Farrer, A. and Spark, J. I. and Scott, D. J. | 1997 | Autologous blood transfusion: the benefits to the patient undergoing abdominal aortic aneurysm repair | J Vasc Nurs | 50 |  |
| **AO5** | Wong, J. C. and Torella, F. and Haynes, S. L. and Dalrymple, K. and Mortimer, A. J. and McCollum, C. N. and Investigators, Atis | 2002 | Autologous versus allogeneic transfusion in aortic surgery: a multicenter randomized clinical trial | Ann Surg | 145 |  |
| **AO6** | Bali, C. and Papakostas, J. and Georgiou, G. and Kouvelos, G. and Avgos, S. and Arnaoutoglou, E. and Papadopoulos, G. and Matsagkas, M. | 2015 | A comparative study of sutured versus bovine pericardium mesh abdominal closure after open abdominal aortic aneurysm repair | Hernia | 40 |  |
| **AO7** | Gold, M. S. and Rockman, C. B. and Riles, T. S. | 1997 | Comparison of lumbar and thoracic epidural narcotics for postoperative analgesia in patients undergoing abdominal aortic aneurysm repair | J Cardiothorac Vasc Anesth | 52 |  |
| **AO8** | Sieunarine, K. and Lawrence-Brown, M. M. and Goodman, M. A. | 1997 | Comparison of transperitoneal and retroperitoneal approaches for infrarenal aortic surgery: early and late results | Cardiovasc Surg | 100 |  |
| **AO9** | Bein, B. and Turowski, P. and Renner, J. and Hanss, R. and Steinfath, M. and Scholz, J. and Tonner, P. H. | 2005 | Comparison of xenon-based anaesthesia compared with total intravenous anaesthesia in high risk surgical patients | Anaesthesia | 39 |  |
| **AO10** | Ball, L. and Pellerano, G. and Corsi, L. and Giudici, N. and Pellegrino, A. and Cannata, D. and Santori, G. and Palombo, D. and Pelosi, P. and Gratarola, A. | 2016 | Continuous epidural versus wound infusion plus single morphine bolus as postoperative analgesia in open abdominal aortic aneurysm repair: a randomized non-inferiority trial | Minerva Anestesiol | 51 |  |
| **AO11** | Webster, S. E. and Smith, J. and Thompson, M. M. and Bell, P. R. and Naylor, A. R. | 2004 | Does the sequence of clamp application during open abdominal aortic aneurysm surgery influence distal embolisation? | Eur J Vasc Endovasc Surg | 40 |  |
| **AO12** | Abdul-Hussien, H. and Hanemaaijer, R. and Verheijen, J. H. and van Bockel, J. H. and Geelkerken, R. H. and Lindeman, J. H. | 2009 | Doxycycline therapy for abdominal aneurysm: Improved proteolytic balance through reduced neutrophil content | J Vasc Surg | 60 |  |
| **AO13** | Wolowczyk, L. and Nevin, M. and Day, A. and Smith, F. C. and Baird, R. N. and Lamont, P. M. | 2005 | The effect of acute normovolaemic haemodilution on the inflammatory response and clinical outcome in abdominal aortic aneurysm repair--results of a pilot trial | Eur J Vasc Endovasc Surg | 36 |  |
| **AO14** | Mahmoud, K. M. and Ammar, A. S. | 2011 | Effect of N-acetylcysteine on cardiac injury and oxidative stress after abdominal aortic aneurysm repair: a randomized controlled trial | Acta Anaesthesiol Scand | 55 |  |
| **AO15** | Baldwin, L. and Henderson, A. and Hickman, P. | 1994 | Effect of postoperative low-dose dopamine on renal function after elective major vascular surgery | Ann Intern Med | 37 |  |
| **AO16** | Pedersen, T. F. | 2019 | Effect of Remote Ischemic Preconditioning on Myocardial Infarction During Open Surgery for Ruptured Abdominal Aortic Aneurysm, A Randomized Controlled Trial | Eur J Vasc Endovasc Surg | 200 |  |
| **AO17** | Valentine, R. J. and Duke, M. L. and Inman, M. H. and Grayburn, P. A. and Hagino, R. T. and Kakish, H. B. and Clagett, G. P. | 1998 | Effectiveness of pulmonary artery catheters in aortic surgery: a randomized trial | J Vasc Surg | 120 |  |
| **AO18** | Shi, C. and Chen, Q. | 2019 | Efficacy of Unibody Bifurcated Endovascular Stent-Graft Repair in the Treatment of Abdominal Aortic Aneurysm | J Coll Physicians Surg Pak | 80 |  |
| **AO19** | Improve Trial Investigators and Powell, J. T. and Sweeting, M. J. and Thompson, M. M. and Ashleigh, R. and Bell, R. and Gomes, M. and Greenhalgh, R. M. and Grieve, R. and Heatley, F. and Hinchliffe, R. J. and Thompson, S. G. and Ulug, P. | 2014 | Endovascular or open repair strategy for ruptured abdominal aortic aneurysm: 30 day outcomes from IMPROVE randomised trial | Brit Med J | 613 |  |
| **AO20** | Greenhalgh, R. M. and Brown, L. C. and Powell, J. T. and Thompson, S. G. and Epstein, D. and Sculpher, M. J. | 2010 | Endovascular versus open repair of abdominal aortic aneurysm | N Engl J Med | 1252 |  |
| **AO21** | Yuceyar, L. and Erolcay, H. and Konukoglu, D. and Bozkurt, A. K. and Aykac, B. | 2004 | Epidural anesthesia may attenuate lipid peroxidation during aorto-femoral surgery | Can J Anesth | 40 |  |
| **AO22** | Lombardo, L. and Ruggia, O. and Crocella, L. and Masoero, G. and Foti, M. and Mambrini, S. and Palombo, D. and Melchiorri, C. and Lupo, M. and Pera, A. | 2009 | Epidural plus general anesthesia vs general anesthesia alone for elective aortic surgery: effects on gastric electrical activity and serum gastrin secretion | Minerva Anestesiol | 34 |  |
| **AO23** | Welch, M. and Newstead, C. G. and Smyth, J. V. and Dodd, P. D. and Walker, M. G. | 1995 | Evaluation of dopexamine hydrochloride as a renoprotective agent during aortic surgery | Ann Vasc Surg | 32 |  |
| **AO24** | Wolowczyk, L. and Nevin, M. and Smith, F. C. and Baird, R. N. and Lamont, P. M. | 2003 | Haemodilutional effect of standard fluid management limits the effectiveness of acute normovolaemic haemodilution in AAA surgery--results of a pilot trial | Eur J Vasc Endovasc Surg | 34 |  |
| **AO25** | Houweling, P. L. and Joosten, W. | 1993 | A haemodynamic comparison of intrathecal morphine and sufentanil supplemented with general anaesthesia for abdominal aortic surgery | Eur J Vasc Surg | 36 |  |
| **AO26** | Fourneau, I. and Sabbe, T. and Daenens, K. and Nevelsteen, A. | 2006 | Hand-assisted laparoscopy versus conventional median laparotomy for aortobifemoral bypass for severe aorto-iliac occlusive disease: a prospective randomised study | Eur J Vasc Endovasc Surg | 36 |  |
| **AO27** | Lottman, P. E. and Laheij, R. J. and Cuypers, P. W. and Bender, M. and Buth, J. | 2004 | Health-related quality of life outcomes following elective open or endovascular AAA repair: a randomized controlled trial | J Endovasc Ther | 76 |  |
| **AO28** | Ragaller, M. and Muller, M. and Bleyl, J. U. and Strecker, A. and Segiet, T. W. and Ellinger, K. and Albrecht, D. M. | 2000 | Hemodynamic effects of hypertonic hydroxyethyl starch 6% solution and isotonic hydroxyethyl starch 6% solution after declamping during abdominal aortic aneurysm repair | Shock | 32 |  |
| **AO29** | Sumi, K. and Iida, H. and Yamaguchi, S. and Fukuoka, N. and Shimabukuro, K. and Dohi, S. | 2008 | Human atrial natriuretic peptide prevents the increase in pulmonary artery pressure associated with aortic unclamping during abdominal aortic aneurysmectomy | J Cardiothorac Vasc Anesth | 45 |  |
| **AO30** | Lederle, F. A. and Wilson, S. E. and Johnson, G. R. and Reinke, D. B. and Littooy, F. N. and Acher, C. W. and Ballard, D. J. and Messina, L. M. and Gordon, I. L. and Chute, E. P. and Krupski, W. C. and Busuttil, S. J. and Barone, G. W. and Sparks, S. and Graham, L. M. and Rapp, J. H. and Makaroun, M. S. and Moneta, G. L. and Cambria, R. A. and Makhoul, R. G. and Eton, D. and Ansel, H. J. and Freischlag, J. A. and Bandyk, D. | 2002 | Immediate repair compared with surveillance of small abdominal aortic aneurysms | N Engl J Med | 1136 |  |
| **AO31** | Bonazzi, M. and Gentile, F. and Biasi, G. M. and Migliavacca, S. and Esposti, D. and Cipolla, M. and Marsicano, M. and Prampolini, F. and Ornaghi, M. and Sternjakob, S. and Tshomba, Y. | 2002 | Impact of perioperative haemodynamic monitoring on cardiac morbidity after major vascular surgery in low risk patients. A randomised pilot trial | Eur J Vasc Endovasc Surg | 100 |  |
| **AO32** | Utoh, J. and Miyauchi, Y. and Goto, H. and Obayashi, H. and Hirata, T. | 1996 | Inflammatory reactions after vascular prosthesis implantation: a comparison of gelatin-sealed and unsealed Dacron prostheses | Surg Today | 50 |  |
| **AO33** | Owczuk, R. and Dylczyk-Sommer, A. and Wojciechowski, J. and Paszkiewicz, M. and Wujtewicz, M. and Stepnowski, P. and Twardowski, P. and Sawicka, W. and Domzalski, M. and Wujtewicz, M. A. | 2016 | The influence of epidural blockade on gut permeability in patients undergoing open surgical repair of abdominal aortic aneurysm | Anestezjol Intens Ter | 70 |  |
| **AO34** | Erkalp, K. and Inan, B. and Abut, Y. and Teker, G. and Basaranoglu, G. and Kalko, Y. and Gumus, F. | 2014 | Interleukin-10 Levels and Clinical Outcome : Comparison of Retroperitoneal versus Transperitoneal Approaches in Infra-Renal Abdominal Aorta Reconstruction | Acta Chir Belg | 100 |  |
| **AO35** | Thompson, J. F. and Mullee, M. A. and Bell, P. R. and Campbell, W. B. and Chant, A. D. and Darke, S. G. and Jamieson, C. W. and Murie, J. and Parvin, S. D. and Perry, M. and Ruckley, C. V. and Wolfe, J. N. and Clyne, C. A. | 1996 | Intraoperative heparinisation, blood loss and myocardial infarction during aortic aneurysm surgery: a Joint Vascular Research Group study | Eur J Vasc Endovasc Surg | 284 |  |
| **AO36** | Li, C. and Li, Y. S. and Xu, M. and Wen, S. H. and Yao, X. and Wu, Y. and Huang, C. Y. and Huang, W. Q. and Liu, K. X. | 2013 | Limb remote ischemic preconditioning for intestinal and pulmonary protection during elective open infrarenal abdominal aortic aneurysm repair: a randomized controlled trial | Anesthesiology | 62 |  |
| **AO37** | Watters, J. M. and Vallerand, A. and Kirkpatrick, S. M. and Abbott, H. E. and Norris, S. and Wells, G. and Barber, G. G. | 2002 | Limited effects of micronutrient supplementation on strength and physical function after abdominal aortic aneurysmectomy | Clin Nutr | 36 |  |
| **AO38** | Ding, R. and McGuinness, C. L. and Burnand, K. G. and Sullivan, E. and Smith, A. | 2005 | Matrix metalloproteinases in the aneurysm wall of patients treated with low-dose doxycycline | Vascular | 56 |  |
| **AO39** | Gogenur, I. and Kucukakin, B. and Jensen, L. P. and Reiter, R. J. and Rosenberg, J. | 2014 | Melatonin reduces cardiac morbidity and markers of myocardial ischemia after elective abdominal aortic aneurism repair: a randomized, placebo-controlled, clinical trial | J Pineal Res | 50 |  |
| **AO40** | Wijnen, M. H. and Roumen, R. M. and Vader, H. L. and Goris, R. J. | 2002 | A multiantioxidant supplementation reduces damage from ischaemia reperfusion in patients after lower torso ischaemia. A randomised trial | Eur J Vasc Endovasc Surg | 42 |  |
| **AO41** | Hynninen, M. S. and Niemi, T. T. and Poyhia, R. and Raininko, E. I. and Salmenpera, M. T. and Lepantalo, M. J. and Railo, M. J. and Tallgren, M. K. | 2006 | N-acetylcysteine for the prevention of kidney injury in abdominal aortic surgery: A randomized, double-blind, placebo-controlled trial | Anesth Analg | 70 |  |
| **AO42** | Kucukakin, B. and Wilhelmsen, M. and Lykkesfeldt, J. and Reiter, R. J. and Rosenberg, J. and Gogenur, I. | 2010 | No Effect of Melatonin to Modify Surgical-Stress Response after Major Vascular Surgery: A Randomised Placebo-controlled trial | Eur J Vasc Endovasc Surg | 52 |  |
| **AO43** | Waters, J. H. and Gottlieb, A. and Schoenwald, P. and Popovich, M. J. and Sprung, J. and Nelson, D. R. | 2001 | Normal saline versus lactated Ringer's solution for intraoperative fluid management in patients undergoing abdominal aortic aneurysm repair: an outcome study | Anesth Analg | 66 |  |
| **AO44** | Elmore, J. R. and Franklin, D. P. and Youkey, J. R. and Oren, J. W. and Frey, C. M. | 1998 | Normothermia is protective during infrarenal aortic surgery | J Vasc Surg | 100 |  |
| **AO45** | Bisgaard, J. and Gilsaa, T. and Ronholm, E. and Toft, P. | 2013 | Optimising stroke volume and oxygen delivery in abdominal aortic surgery: a randomised controlled trial | Acta Anaesth Scand | 64 |  |
| **AO46** | Soulez, G. and Therasse, E. and Monfared, A. A. and Blair, J. F. and Choiniere, M. and Elkouri, S. and Beaudoin, N. and Giroux, M. F. and Cliche, A. and Lelorier, J. and Oliva, V. L. | 2005 | Pain and quality of life assessment after endovascular versus open repair of abdominal aortic aneurysms in patients at low risk | J Vasc Interv Radiol | 40 |  |
| **AO47** | Barakat, H. M. and Shahin, Y. and Khan, J. A. and McCollum, P. T. and Chetter, I. C. | 2016 | Preoperative Supervised Exercise Improves Outcomes After Elective Abdominal Aortic Aneurysm Repair: A Randomized Controlled Trial | Ann Surg | 124 |  |
| **AO48** | Muehling, B. M. and Halter, G. and Lang, G. and Schelzig, H. and Steffen, P. and Wagner, F. and Meierhenrich, R. and Sunder-Plassmann, L. and Orend, K. H. | 2008 | Prospective randomized controlled trial to evaluate "fast-track" elective open infrarenal aneurysm repair | Langenbecks Arch Surg | 82 |  |
| **AO49** | Friedman, S. G. and Sowerby, S. A. and Del Pin, C. A. and Scher, L. A. and Tortolani, A. J. | 1996 | A prospective randomized study of abdominal aortic surgery without postoperative nasogastric decompression | Cardiovasc Surg | 80 |  |
| **AO50** | Pillai, J. and Britz, R. and Cluver, M. D. and Candy, G. P. and Abdool-Carrim, A. T. and Veller, M. G. | 2008 | Proximal suture line support in open abdominal aortic aneurysm repair: a comparative study | Eur J Vasc Endovasc Surg | 40 |  |
| **AO51** | Moxon, J. V. and Rowbotham, S. E. and Pinchbeck, J. L. and Lazzaroni, S. M. and Morton, S. K. and Moran, C. S. and Quigley, F. and Jenkins, J. S. and Reid, C. M. and Cavaye, D. and Jaeggi, R. and Golledge, J. | 2020 | A Randomised Controlled Trial Assessing the Effects of Peri-operative Fenofibrate Administration on Abdominal Aortic Aneurysm Pathology: Outcomes From the FAME Trial | Eur J Vasc Endovasc Surg | 43 |  |
| **AO52** | Hinchliffe, R. J. and Bruijstens, L. and MacSweeney, S. T. and Braithwaite, B. D. | 2006 | A randomised trial of endovascular and open surgery for ruptured abdominal aortic aneurysm - results of a pilot study and lessons learned for future studies | Eur J Vasc Endovasc Surg | 32 |  |
| **AO53** | Mahmood, A. and Gosling, P. and Vohra, R. K. | 2007 | Randomized clinical trial comparing the effects on renal function of hydroxyethyl starch or gelatine during aortic aneurysm surgery | Br J Surg | 62 |  |
| **AO54** | Mercer, K. G. and Spark, J. I. and Berridge, D. C. and Kent, P. J. and Scott, D. J. | 2004 | Randomized clinical trial of intraoperative autotransfusion in surgery for abdominal aortic aneurysm | Br J Surg | 81 |  |
| **AO55** | Bevis, P. M. and Windhaber, R. A. and Lear, P. A. and Poskitt, K. R. and Earnshaw, J. J. and Mitchell, D. C. | 2010 | Randomized clinical trial of mesh versus sutured wound closure after open abdominal aortic aneurysm surgery | Br J Surg | 85 |  |
| **AO56** | Becquemin, J. P. and Pillet, J. C. and Lescalie, F. and Sapoval, M. and Goueffic, Y. and Lermusiaux, P. and Steinmetz, E. and Marzelle, J. and trialists, A. C. E. | 2011 | A randomized controlled trial of endovascular aneurysm repair versus open surgery for abdominal aortic aneurysms in low- to moderate-risk patients | J Vasc Surg | 306 |  |
| **AO57** | Funk, D. J. and HayGlass, K. T. and Koulack, J. and Harding, G. and Boyd, A. and Brinkman, R. | 2015 | A randomized controlled trial on the effects of goal-directed therapy on the inflammatory response open abdominal aortic aneurysm repair | Crit Care | 40 |  |
| **AO58** | Rahman, M. N. and Khan, J. A. and Mazari, F. A. and Mockford, K. and McCollum, P. T. and Chetter, I. C. | 2011 | A randomized placebo controlled trial of the effect of preoperative statin use on matrix metalloproteinases and tissue inhibitors of matrix metalloproteinases in areas of low and peak wall stress in patients undergoing elective open repair of abdominal aortic aneurysm | Ann Vasc Surg | 40 |  |
| **AO59** | Cuypers, P. W. and Gardien, M. and Buth, J. and Peels, C. H. and Charbon, J. A. and Hop, W. C. | 2001 | Randomized study comparing cardiac response in endovascular and open abdominal aortic aneurysm repair | Br J Surg | 76 |  |
| **AO60** | Rittoo, D. and Gosling, P. and Burnley, S. and Bonnici, C. and Millns, P. and Simms, M. H. and Smith, S. R. and Vohra, R. K. | 2004 | Randomized study comparing the effects of hydroxyethyl starch solution with Gelofusine on pulmonary function in patients undergoing abdominal aortic aneurysm surgery | Br J Anaesth | 40 |  |
| **AO61** | Prinssen, M. and Verhoeven, E. L. and Buth, J. and Cuypers, P. W. and van Sambeek, M. R. and Balm, R. and Buskens, E. and Grobbee, D. E. and Blankensteijn, J. D. and Dutch Randomized Endovascular Aneurysm Management Trial, Group | 2004 | A randomized trial comparing conventional and endovascular repair of abdominal aortic aneurysms | New Engl J Med | 345 |  |
| **AO62** | Clagett, G. P. and Valentine, R. J. and Jackson, M. R. and Mathison, C. and Kakish, H. B. and Bengtson, T. D. | 1999 | A randomized trial of intraoperative autotransfusion during aortic surgery | J Vasc Surg | 100 |  |
| **AO63** | Robinson, J. and Nawaz, S. and Beard, J. D. and Joint Vasc Res, Grp | 2000 | Randomized, multicentre, double-blind, placebo-controlled trial of the use of aprotinin in the repair of ruptured abdominal aortic aneurysm | Br J Surg | 77 |  |
| **AO64** | Mouton, R. and Pollock, J. and Soar, J. and Mitchell, D. C. and Rogers, C. A. | 2015 | Remote ischaemic preconditioning versus sham procedure for abdominal aortic aneurysm repair: an external feasibility randomized controlled trial | Trials | 69 |  |
| **AO65** | Walsh, S. R. and Sadat, U. and Boyle, J. R. and Tang, T. Y. and Lapsley, M. and Norden, A. G. and Gaunt, M. E. | 2010 | Remote ischemic preconditioning for renal protection during elective open infrarenal abdominal aortic aneurysm repair: randomized controlled trial | Vasc Endovascular Surg | 40 |  |
| **AO66** | Ali, Z. A. and Callaghan, C. J. and Lim, E. and Ali, A. A. and Nouraei, S. A. and Akthar, A. M. and Boyle, J. R. and Varty, K. and Kharbanda, R. K. and Dutka, D. P. and Gaunt, M. E. | 2007 | Remote ischemic preconditioning reduces myocardial and renal injury after elective abdominal aortic aneurysm repair: a randomized controlled trial | Circulation | 82 |  |
| **AO67** | Piljic, D. and Petricevic, M. and Piljic, D. and Ksela, J. and Robic, B. and Klokocovnik, T. | 2016 | Restrictive versus Standard Fluid Regimen in Elective Minilaparotomy Abdominal Aortic Repair-Prospective Randomized Controlled Trial | Thorac Cardiovasc Surg | 60 |  |
| **AO68** | Arya, N. and Muhammad Anees, Sharif and Lau, L. L. and Lee, B. and Hannon, R. J. and Young, I. S. and Chee Voon, Soong | 2009 | Retroperitoneal repair of abdominal aortic aneurysm reduces bowel dysfunction | Vasc Endovascular Surg | 34 |  |
| **AO69** | Hayashi, Y. and Ohtani, M. and Sawa, Y. and Hiraishi, T. and Akedo, H. and Kobayashi, Y. and Matsuda, H. | 2003 | Synthetic human alpha-atrial natriuretic peptide improves the management of postoperative hypertension and renal dysfunction after the repair of abdominal aortic aneurysm | J Cardiovasc Pharmacol | 50 |  |
| **AO70** | Monaco, F. and Nardelli, P. and Pasin, L. and Barucco, G. and Mattioli, C. and Di Tomasso, N. and Dalessandro, G. and Giardina, G. and Landoni, G. and Chiesa, R. and Zangrillo, A. | 2020 | Tranexamic acid in open aortic aneurysm surgery: a randomised clinical trial | Brit J Anaesth | 100 |  |
| **AO71** | Sicard, G. A. and Reilly, J. M. and Rubin, B. G. and Thompson, R. W. and Allen, B. T. and Flye, M. W. and Schechtman, K. B. and Young-Beyer, P. and Weiss, C. and Anderson, C. B. | 1995 | Transabdominal versus retroperitoneal incision for abdominal aortic surgery: report of a prospective randomized trial | J Vasc Surg | 145 |  |
| **AO72** | Boker, A. and Haberman, C. J. and Girling, L. and Guzman, R. P. and Louridas, G. and Tanner, J. R. and Cheang, M. and Maycher, B. W. and Bell, D. D. and Doak, G. J. | 2004 | Variable ventilation improves perioperative lung function in patients undergoing abdominal aortic aneurysmectomy | Anesthesiology | 41 |  |
| **AO73** | Duffy, M. J. and O'Kane, C. M. and Stevenson, M. and Young, I. S. and Harkin, D. W. and Mullan, B. A. and McAuley, D. F. | 2015 | A randomized clinical trial of ascorbic acid in open abdominal aortic aneurysm repair | Intens Care Med Exp | 31 |  |
| **AO74** | Dewulf, M. and Muysoms, F. and Vierendeels, T. and Huyghe, M. and Miserez, M. and Ruppert, M. and Tollens, T. and van Bergen, L. and Berrevoet, F. and Detry, O. | 2022 | Prevention of incisional hernias by prophylactic mesh-augmented reinforcement of midline laparotomies for abdominal aortic aneurysm treatment: five-year follow-up of a randomized controlled trial | Ann Surg | 120 |  |
| **AO75** | Eid, M. A. and Barry, M. J. and Tang, G. L. and Henke, P. K. and Johanning, J. M. and Tzeng, E. and Scali, S. T. and Stone, D. H. and Suckow, B. D. and Lee, E. S. and Arya, S. and Brooke, B. S. and Nelson, P. R. and Spangler, E. L. and Murebee, L. and Dosluoglu, H. H. and Raffetto, J. D. and Kougais, P. and Brewster, L. P. and Alabi, O. and Dardik A. and Halpern V.J. and O'Connell J.B. and Ihnat D.M. and Zhou W. and Sirovich B.E. and Metha K. and Moore K.O. and Voorhees A. and Goodney P.P. and Preferences for Open Versus Endovascular Repair of Abdominal Aortic Aneurysm (PROVE-AAA) Study Team | 2022 | Effect of a decision aid on agreement between patient preferences and repair type for abdominal aortic aneurysm: a randomized clinical trial | JAMA Surg | 118 |  |
| **AO76** | Hakovirta, H. and Jalkanen, J. and Saimanen, E. and Kukkonen, T. and Romsi, P. and Suominen, V. and Vikatmaa, L. and Valtonen, M. and Karvonen, M. K. and Venermo, M. and INFORAAA Study Group | 2022 | Induction of CD73 prevents death after emergency open aortic surgery for a ruptured abdominal aortic aneurysm: a randomized, double-blind | Sci Rep-UK | 40 |  |
| **AO77** | Honig, S. and Diener, H. and Kölbel, T. and Reinpold, W. and Zapf, A. and Bibiza-Freiwald, E. and Debus, E. S., for AIDA study group | 2022 | Abdominal incision defect following AAA-surgery (AIDA): 2-year results of prophylactic onlay-mesh augmentation in a multicentre, double-blind, randomised controlled trial | Updates Surg | 104 |  |

| **Amputation** | | | | | |
| --- | --- | --- | --- | --- | --- |
| **Study identifier** | **Authors** | **Year** | **Title** | **Journal** | **Number of trial participants** |
| **AM1** | Mann, R. A. and Bisset, W. I. | 1983 | Anaesthesia for lower limb amputation. A comparison of spinal analgesia and general anaesthesia in the elderly | Anaesthesia | 60 |
| **AM2** | Sonne-Holm, S. and Boeckstyns, M. and Menck, H. and Sinding, A. and Leicht, P. and Dichmann, O. and Prag, J. B. and Baekgaard, N. and Ostri, P. and GÃ¸trik, J. K. | 1985 | Prophylactic antibiotics in amputation of the lower extremity for ischemia. A placebo-controlled, randomized trial of cefoxitin | J Bone Joint Surg Am | 152 |
| **AM3** | Friis H. | 1987 | Penicillin G versus cefuroxime for prophylaxis in lower limb amputation | Acta Orthop Scand | 457 |
| **AM4** | Fisher, D. F., Jr. and Clagett, G. P. and Fry, R. E. and Humble, T. H. and Fry, W. J. | 1988 | One-stage versus two-stage amputation for wet gangrene of the lower extremity: a randomized study | J Vasc Surg | 47 |
| **AM5** | Norlin, R. and Frydén, A. and Nilsson, L. and Anséhn, S. | 1990 | Short-term cefotaxime prophylaxis reduces the failure rate in lower limb amputations | Acta Orthop Scand | 38 |
| **AM6** | Dormandy, J. and Belcher, G. and Broos, P. and Eikelboom, B. and Laszlo, G. and Konrad, P. and Moggi, L. and Mueller, U. | 1994 | Prospective study of 713 below-knee amputations for ischaemia and the effect of a prostacyclin analogue on healing. Hawaii Study Group | Br J Surg | 713 |
| **AM7** | Nikolajsen, L. and Ilkjaer, S. and Jensen, T. S. | 1998 | Effect of preoperative extradural bupivacaine and morphine on stump sensation in lower limb amputees | Brit J Anaesth | 60 |
| **AM8** | Hayes, C. and Armstrong-Brown, A. and Burstal, R. | 2004 | Perioperative intravenous ketamine infusion for the prevention of persistent post-amputation pain: a randomized, controlled trial | Anaesth Intens Care | 45 |
| **AM9** | Deutsch, A. and English, R. D. and Vermeer, T. C. and Murray, P. S. and Condous, M. | 2005 | Removable rigid dressings versus soft dressings: a randomized, controlled study with dysvascular, trans-tibial amputees | Prosthet Orthot Int | 50 |
| **AM10** | Nikolajsen, L. and Finnerup, N. B. and Kramp, S. and Vimtrup, A. S. and Keller, J. and Jensen, T. S. | 2006 | A randomized study of the effects of gabapentin on postamputation pain | Anesthesiology | 46 |
| **AM11** | Reuben, S. S. and Raghunathan, K. and Roissing, S. | 2006 | Evaluating the analgesic effect of the perioperative perineural infiltration of bupivacaine and clonidine at the site of injury following lower extremity amputation | Acute pain | 80 |
| **AM12** | Lastoria, S. and Rollo, H. A. and Yoshida, W. B. and Giannini, M. and Moura, R. and Maffei, F. H. A. | 2006 | Prophylaxis of deep-vein thrombosis after lower extremity amputation. Comparison of low molecular weight heparin with unfractionated heparin | Acta Cirurgica Brasileira | 75 |
| **AM13** | Choksy, S. A. and Lee Chong, P. and Smith, C. and Ireland, M. and Beard, J. | 2006 | A randomised controlled trial of the use of a tourniquet to reduce blood loss during transtibial amputation for peripheral arterial disease | Eur J Vasc Endovasc Surg | 64 |
| **AM14** | Wilson, J. A. and Nimmo, A. F. and Fleetwood-Walker, S. M. and Colvin, L. A. | 2008 | A randomised double blind trial of the effect of pre-emptive epidural ketamine on persistent pain after lower limb amputation | Pain | 53 |
| **AM15** | Karanikolas, M. and Aretha, D. and Tsolakis, I. and Monantera, G. and Kiekkas, P. and Papadoulas, S. and Swarm, R. A. and Filos, K. S. | 2011 | Optimized perioperative analgesia reduces chronic phantom limb pain intensity, prevalence, and frequency: a prospective, randomized, clinical trial | Anesthesiology | 65 |
| **AM16** | Hussien, R. M. and Ibrahim, D. A. and Abdelnaby, I. G. H. | 2018 | Ultrasound-guided sciatic nerve block in below knee amputation surgery: Sub gluteal versus popliteal approach | Open J Anesthesiol | 56 |
| **AM17** | Bosanquet, D. C. and Ambler, G. K. and Waldron, C. A. and Thomas-Jones, E. and Brookes-Howell, L. and Kelson, M. and Pickles, T. and Harris, D. and Milosevic, S. and Fitzsimmons, D. and Saxena, N. and Twine, C. P. | 2019 | Perineural local anaesthetic catheter after major lower limb amputation trial (PLACEMENT): results from a randomised controlled feasibility trial | BMJ Open | 50 |
| **AM18** | Buchheit, T. and Hsia, H. J. and Cooter, M. and Shortell, C. and Kent, M. and McDuffie, M. and Shaw, A. and Buckenmaier, C. T. and Van de Ven, T. | 2019 | The impact of surgical amputation and valproic acid on pain and functional trajectory: Results from the Veterans Integrated Pain Evaluation Research (VIPER) randomized, double-blinded placebo-controlled trial | Pain Med | 128 |
| **AM19** | Thompson, J. P. and Bowrey, S. and Viskaduraki, M. and Nath, M. | 2020 | Randomised placebo-controlled trial of continuous sciatic or posterior tibial nerve blockade on pain after major lower limb amputation | Br J Anaesth | 90 |
| **AM20** | Ilfeld, B. M. and Khatibi, B. and Maheshwari, K. and Madison, S. J. and Ali Sakr Esa, W. and Mariano, E. R. and Kent, M. L. and Hanling, S. and Sessler, D. I. and Eisenach, J. C. and Cohen, S. P. and Mascha, E. J. and Yang, D. and Padwal, J. A. and Turan, A. and PAINfRE Investigators | 2021 | Immediate effects of a continuous peripheral nerve block on postamputation phantom and residual limb pain: secondary outcomes from a multicenter randomized controlled clinical trial | Anesth analg | 144 |
| **AM21** | Hunt, W. and Nath, M. and Bowrey, S. and Colvin, L. and Thompson, J. P. | 2023 | Effect of a continuous perineural levobupivacaine infusion on pain after major lower limb amputation: a randomised double-blind placebo-controlled trial | BMJ Open | 81 |
| **AM22** | Lee, J. V. and Engel, C. and Tay, S. and DeSilva, G. and Desai, K. and Cashin, J. and Semenkovich, C. F. and Zayed, M. A. | 2022 | Impact of n-acetyl-cysteine on ischemic stumps following major lower extremity amputation: a pilot randomized clinical trial | Ann Surg | 33 |
| **AM23** | Souroullas, P. and Barnes, R. and Carradice, D. and Smith, G. and Huang, C. and Chetter, I. | 2022 | Extended-course antibiotic prophylaxis in lower limb amputation: randomized clinical trial | Br J Surg | 152 |

| **Bypass** |  |  |  |  |  |
| --- | --- | --- | --- | --- | --- |
| **Study identifier** | **Authors** | **Year** | **Title** | **Journal** | **Number of trial participants** |
| **B1** | Reijnen, Mmpj and van Walraven, L. A. and Fritschy, W. M. and Lensvelt, M. M. A. and Zeebregts, C. J. and Lemson, M. S. and Wikkeling, O. R. M. and Smeets, L. and Holewijn, S. | 2017 | 1-Year Results of a Multicenter Randomized Controlled Trial Comparing Heparin-Bonded EndoluminalÂ to Femoropopliteal Bypass | JACC Cardiovasc Interv | 129 |
| **B2** | de Donato, G. and Gussoni, G. and de Donato, G. and Cao, P. and Setacci, C. and Pratesi, C. and Mazzone, A. and Ferrari, M. and Veglia, F. and Bonizzoni, E. and Settembrini, P. and Ebner, H. and Martino, A. and Palombo, D. | 2007 | Acute limb ischemia in elderly patients: can iloprost be useful as an adjuvant to surgery? Results from the ILAILL study | Eur J Vasc Endovasc Surg | 192 |
| **B3** | Pierce, E. T. and Pomposelli, F. B., Jr. and Stanley, G. D. and Lewis, K. P. and Cass, J. L. and LoGerfo, F. W. and Gibbons, G. W. and Campbell, D. R. and Freeman, D. V. and Halpern, E. F. and Bode, R. H., Jr. | 1997 | Anesthesia type does not influence early graft patency or limb salvage rates of lower extremity arterial bypass | J Vasc Surg | 423 |
| **B4** | van der Zaag, E. S. and Legemate, D. A. and Prins, M. H. and Reekers, J. A. and Jacobs, M. J. | 2004 | Angioplasty or bypass for superficial femoral artery disease? - A randomised controlled trial | Eur J Vasc Endovasc Surg | 56 |
| **B5** | Wilson, Y. G. and Davies, A. H. and Currie, I. C. and McGrath, C. and Morgan, M. and Baird, R. N. and Lamont, P. M. | 1996 | Angioscopically-assisted in situ saphenous vein bypass for infrainguinal revascularisation | Eur J Vasc Endovasc Surg | 47 |
| **B6** | Worning, A. M. and Frimodt-Moller, N. and Ostri, P. and Nilsson, T. and Hojholdt, K. and Frimodt-Moller, C. | 1986 | Antibiotic prophylaxis in vascular reconstructive surgery: a double-blind placebo-controlled study | J Antimicrob Chemother | 155 |
| **B7** | McCollum, C. and Alexander, C. and Kenchington, G. and Franks, P. J. and Greenhalgh, R. | 1991 | Antiplatelet drugs in femoropopliteal vein bypasses: a multicenter trial | J Vasc Surg | 549 |
| **B8** | Goldman, M. D. and Simpson, D. and Hawker, R. J. and Norcott, H. C. and McCollum, C. N. | 1983 | Aspirin and dipyridamole reduce platelet deposition on prosthetic femoro-popliteal grafts in man | Ann Surg | 47 |
| **B9** | Adam, D. J. and Beard, J. D. and Cleveland, T. and Bell, J. and Bradbury, A. W. and Forbes, J. F. and Fowkes, F. G. and Gillepsie, I. and Ruckley, C. V. and Raab, G. and Storkey, H. | 2005 | Bypass versus angioplasty in severe ischaemia of the leg (BASIL): multicentre, randomised controlled trial | Lancet | 452 |
| **B10** | Garcia, S. and Rector, T. S. and Zakharova, M. and Herrmann, R. R. and Adabag, S. and Bertog, S. and Sandoval, Y. and Santilli, S. and Brilakis, E. S. and McFalls, E. O. | 2016 | Cardiac Remote Ischemic Preconditioning Prior to Elective Vascular Surgery (CRIPES): A Prospective, Randomized, Sham-Controlled PhaseÂ II Clinical Trial | J Am Heart Assoc | 201 |
| **B11** | Baumgartner, I. and Norgren, L. and Fowkes, F. G. R. and Mulder, H. and Patel, M. R. and Berger, J. S. and Jones, W. S. and Rockhold, F. W. and Katona, B. G. and Mahaffey, K. and Hiatt, W. R. | 2018 | Cardiovascular Outcomes After LowerÂ Extremity Endovascular or SurgicalÂ Revascularization: The EUCLID Trial | J Am Coll Cardiol | 1738 |
| **B12** | Zilla, P. and Deutsch, M. and Meinhart, J. and Puschmann, R. and Eberl, T. and Minar, E. and Dudczak, R. and Lugmaier, H. and Schmidt, P. and Noszian, I. and et al. | 1994 | Clinical in vitro endothelialization of femoropopliteal bypass grafts: an actuarial follow-up over three years | J Vasc Surg | 49 |
| **B13** | Monaco, M. and Di Tommaso, L. and Pinna, G. B. and Lillo, S. and Schiavone, V. and Stassano, P. | 2012 | Combination therapy with warfarin plus clopidogrel improves outcomes in femoropopliteal bypass surgery patients | J Vasc Surg | 341 |
| **B14** | Johnson, W. C. and Lee, K. K. | 1999 | Comparative evaluation of externally supported Dacron and polytetrafluoroethylene prosthetic bypasses for femorofemoral and axillofemoral arterial reconstructions. Veterans Affairs Cooperative Study #141 | J Vasc Surg | 419 |
| **B15** | Johnson, W. C. and Lee, K. K. | 2000 | A comparative evaluation of polytetrafluoroethylene, umbilical vein, and saphenous vein bypass grafts for femoral-popliteal above-knee revascularization: a prospective randomized Department of Veterans Affairs cooperative study | J Vasc Surg | 752 |
| **B16** | Mohammadi Tofigh, A. and Warnier De Wailly, G. and Rhissassi, B. | 2007 | Comparing vein with collagen impregnated woven polyester prosthesis in above-knee femoropopliteal bypass grafting | Int Surg J | 103 |
| **B17** | Miller, A. and Marcaccio, E. J. and Tannenbaum, G. A. and Kwolek, C. J. and Stonebridge, P. A. and Lavin, P. T. and Gibbons, G. W. and Pomposelli, F. B., Jr. and Freeman, D. V. and Campbell, D. R. and et al. | 1993 | Comparison of angioscopy and angiography for monitoring infrainguinal bypass vein grafts: results of a prospective randomized trial | J Vasc Surg | 250 |
| **B18** | Kapfer, X. and Meichelboeck, W. and Groegler, F. M. | 2006 | Comparison of carbon-impregnated and standard ePTFE prostheses in extra-anatomical anterior tibial artery bypass: a prospective randomized multicenter study | Eur J Vasc Endovasc Surg | 265 |
| **B19** | Lumsden, A. B. and Morrissey, N. J. | 2015 | Comparison of Safety and Primary Patency Between the FUSION BIOLINE Heparin-Coated Vascular Graft and EXXCEL Soft ePTFE (FINEST) Trial Co-investigators. Randomized controlled trial comparing the safety and efficacy between the FUSION BIOLINE heparin-coated vascular graft and the standard expanded polytetrafluoroethylene graft for femoropopliteal bypass (vol 61, pg 703, 2015) | J Vasc Surg | 209 |
| **B20** | Davidovic, L. and Jakovljevic, N. and Radak, D. and Dragas, M. and Ilic, N. and Koncar, I. and Markovic, D. | 2010 | Dacron or ePTFE graft for above-knee femoropopliteal bypass reconstruction. A bi-centre randomised study | Vasa | 85 |
| **B21** | Laurila, K. and LepÃ¤ntalo, M. and Teittinen, K. and Kantonen, I. and Forssell, C. and Vilkko, P. and Nielsen, O. M. and Railo, M. and Lehtola, A. | 2004 | Does an adjuvant AV-fistula improve the patency of a femorocrural PTFE bypass with distal vein cuff in critical leg ischaemia?--a prospective randomised multicentre trial | Eur J Vasc Endovasc Surg | 59 |
| **B22** | Katz, S. G. and Kohl, R. D. and Rutherford, R. B. and Krupski, W. C. and Tawes, R. | 1998 | Does dextran 40 improve the early patency of autogenous infrainguinal bypass grafts? | J Vasc Surg | 244 |
| **B23** | Kreienberg, P. B. and Darling, R. C., 3rd and Chang, B. B. and Champagne, B. J. and Paty, P. S. and Roddy, S. P. and Lloyd, W. E. and Ozsvath, K. J. and Shah, D. M. | 2002 | Early results of a prospective randomized trial of spliced vein versus polytetrafluoroethylene graft with a distal vein cuff for limb-threatening ischemia | J Vasc Surg | 38 |
| **B24** | JakubseviÄienÄ—, E. and Vasiliauskas, D. and VeliÄka, L. and Kubilius, R. and MilinaviÄienÄ—, E. and VenclovienÄ—, J. | 2014 | Effectiveness of a new exercise program after lower limb arterial blood flow surgery in patients with peripheral arterial disease: a randomized clinical trial | Int J Environ Res Public Health | 117 |
| **B25** | Charvin, M. and Longeras, F. and Jouve, P. and Cherprenet, A. L. and Futier, E. and Pereira, B. and DualÃ©, C. | 2020 | Effects of adding a combined femoral and sciatic nerve block with levobupivacaine and clonidine to general anaesthesia in femoropopliteal bypass surgery: A randomised, double-blind, controlled trial | Eur J Anaesthesiol | 47 |
| **B26** | The Iloprost Bypass International Study Group | 1996 | Effects of perioperative iloprost on patency of femorodistal bypass grafts | Eur J Vasc Endovasc Surg | 517 |
| **B27** | Totić, D. and Ðurović Sarajlić, V. and Vranić, H. and Hadžimehmedagić, A. and Rustempašić, N. and Djedović, M. and Vukas, H. and Ahmetašević, A. | 2020 | Endovascular or open surgical treatment of high-risk patients with infrainguinal peripheral arterial disease and critical limb ischemia | Med Glas (Zenica) | 80 |
| **B28** | Schouten, O. and Hoedt, M. T. and Wittens, C. H. and Hop, W. C. and van Sambeek, M. R. and van Urk, H. | 2005 | End-to-end versus end-to-side distal anastomosis in femoropopliteal bypasses; results of a randomized multicenter trial | Eur J Vasc Endovasc Surg | 274 |
| **B29** | Förster, J. G. and Niemi, T. T. and Aromaa, U. and Neuvonen, P. J. and Seppälä, T. A. and Rosenberg, P. H. | 2003 | Epinephrine added to a lumbar epidural infusion of a small-dose ropivacaine-fentanyl mixture after arterial bypass surgery of the lower extremities | Acta Anaesthesiol Scand | 50 |
| **B30** | Mann, M. J. and Whittemore, A. D. and Donaldson, M. C. and Belkin, M. and Conte, M. S. and Polak, J. F. and Orav, E. J. and Ehsan, A. and Dell'Acqua, G. and Dzau, V. J. | 1999 | Ex-vivo gene therapy of human vascular bypass grafts with E2F decoy: the PREVENT single-centre, randomised, controlled trial | Lancet | 41 |
| **B31** | Solakovic, E. and Totic, D. and Solakovic, S. | 2008 | Femoro-popliteal bypass above knee with saphenous vein vs synthetic graft | BJBMS | 121 |
| **B32** | Darke, S. and Lamont, P. and Chant, A. and Barros D'Sa, A. and Clyne, C. and Harris, P. and Ruckley, C. V. and Bell, P. | 1989 | Femoro-popliteal versus femoro-distal bypass grafting for limb salvage in patients with an "isolated" popliteal segment | Eur J Vasc Surg | 59 |
| **B33** | Eickhoff, J. H. and Broome, A. and Ericsson, B. F. and Buchardt Hansen, H. J. and Kordt, K. F. and Mouritzen, C. and Kvernebo, K. and Norgren, L. and Rostad, H. and Trippestad, A. | 1987 | Four years' results of a prospective, randomized clinical trial comparing polytetrafluoroethylene and modified human umbilical vein for below-knee femoropopliteal bypass | J Vasc Surg | 105 |
| **B34** | Johnson, W. C. and Williford, W. O. and Corson, J. D. and Padberg, F. T., Jr. | 2004 | Hemorrhagic complications during long-term postoperative warfarin administration in patients undergoing lower extremity arterial bypass surgery | Vascular | 831 |
| **B35** | Devine, C. and Hons, B. and McCollum, C. | 2001 | Heparin-bonded Dacron or polytetrafluoroethylene for femoropopliteal bypass grafting: a multicenter trial | J Vasc Surg | 209 |
| **B36** | Scharn, D. M. and Dirven, M. and Barendregt, W. B. and Boll, A. P. and Roelofs, D. and van der Vliet, J. A. | 2008 | Human umbilical vein versus heparin-bonded polyester for femoro-popliteal bypass: 5-year results of a prospective randomized multicentre trial | Eur J Vasc Endovasc Surg | 129 |
| **B37** | Smith, F. C. and Tsang, G. M. and Watson, H. R. and Shearman, C. P. | 1992 | Iloprost reduces peripheral resistance during femoro-distal reconstruction | Eur J Vasc Surg | 33 |
| **B38** | Carpenter, J. P. and Tomaszewski, J. E. | 1997 | Immunosuppression for human saphenous vein allograft bypass surgery: a prospective randomized trial | J Vasc Surg | 40 |
| **B39** | Moody, A. P. and Edwards, P. R. and Harris, P. L. | 1992 | In situ versus reversed femoropopliteal vein grafts: long-term follow-up of a prospective, randomized trial | Br J Surg | 226 |
| **B40** | Watelet, J. and Cheysson, E. and Poels, D. and Menard, J. F. and Papion, H. and Saour, N. and Testart, J. | 1987 | In situ versus reversed saphenous vein for femoropopliteal bypass: a prospective randomized study of 100 cases | Ann Vasc Surg | 102 |
| **B41** | Bearn, P. E. and McCollum, C. N. and Greenhalgh, R. M. | 1993 | The influence of collagen and albumen presealants on knitted Dacron grafts | Eur J Vasc Surg | 36 |
| **B42** | Donaldson, D. R. and Salter, M. C. P. and Kester, R. C. | 1985 | The influence of platelet inhibition on the patency of femoro-popliteal Dacron bypass grafts | Vasc Surg | 65 |
| **B43** | Swedenborg, J. and Nydahl, S. and Egberg, N. | 1996 | Low molecular mass heparin instead of unfractionated heparin during infrainguinal bypass surgery | Eur J Vasc Endovasc Surg | 314 |
| **B44** | Logason, K. and Bergqvist, D. | 2001 | Low molecular weight heparin (enoxaparin) versus dextran in the prevention of early occlusion following arterial bypass surgery distal to the groin | Eur J Vasc Endovasc Surg | 200 |
| **B45** | Edmondson, R. A. and Cohen, A. T. and Das, S. K. and Wagner, M. B. and Kakkar, V. V. | 1994 | Low-molecular weight heparin versus aspirin and dipyridamole after femoropopliteal bypass grafting | Lancet | 95 |
| **B46** | van Dijk, L. C. and van Urk, H. and du Bois, N. A. and Yo, T. I. and Koning, J. and Jansen, W. B. and Wittens, C. H. | 1995 | A new "closed" in situ vein bypass technique results in a reduced wound complication rate | Eur J Vasc Endovasc Surg | 80 |
| **B47** | Chiang, N. and Rodda, O. A. and Sleigh, J. and Vasudevan, T. | 2017 | Perioperative warming, oxygen, and Ilomedin on oxygenation and healing in infrainguinal bypass surgery | J Surg Res | 148 |
| **B48** | Glickman, M. and Gheissari, A. and Money, S. and Martin, J. and Ballard, J. L. and CoSeal Multicenter Vascular, Surger | 2002 | A polymeric sealant inhibits anastomotic suture hole bleeding more rapidly than gelfoam/thrombin - Results of a randomized controlled trial | Arch Surg | 130 |
| **B49** | Linni, K. and Aspalter, M. and Mader, N. and Butturini, E. and Ugurluoglu, A. and Granat, S. and Hitzl, W. and Holzenbein, T. | 2012 | Preoperative duplex vein mapping (DVM) reduces costs in patients undergoing infrainguinal bypass surgery: results of a prospective randomised study | Eur J Vasc Endovasc Surg | 101 |
| **B50** | Cook, P. T. and Davies, M. J. and Cronin, K. D. and Moran, P. | 1986 | A prospective randomised trial comparing spinal anaesthesia using hyperbaric cinchocaine with general anaesthesia for lower limb vascular surgery | Anaesth Intens Care | 87 |
| **B51** | Hamsho, A. and Nott, D. and Harris, P. L. | 1999 | Prospective randomised trial of distal arteriovenous fistula as an adjunct to femoro-infrapopliteal PTFE bypass | Eur J Vasc Endovasc Surg | 57 |
| **B52** | te Slaa, A. and Dolmans, Dejgj and Ho, G. H. and Mulder, P. G. H. and van der Waal, J. C. H. and de Groot, H. G. W. and van der Laan, L. | 2011 | Prospective Randomized Controlled Trial to Analyze the Effects of Intermittent Pneumatic Compression on Edema Following Autologous Femoropopliteal Bypass Surgery | World J Surg | 125 |
| **B53** | Wengerter, K. R. and Veith, F. J. and Gupta, S. K. and Goldsmith, J. and Farrell, E. and Harris, P. L. and Moore, D. and Shanik, G. | 1991 | Prospective randomized multicenter comparison of in situ and reversed vein infrapopliteal bypasses | J Vasc Surg | 108 |
| **B54** | Robinson, B. I. and Fletcher, J. P. and Tomlinson, P. and Allen, R. D. and Hazelton, S. J. and Richardson, A. J. and Stuchbery, K. | 1999 | A prospective randomized multicentre comparison of expanded polytetrafluoroethylene and gelatin-sealed knitted Dacron grafts for femoropopliteal bypass | Cardiovasc Surg | 51 |
| **B55** | Ballotta, E. and Renon, L. and Toffano, M. and Da Giau, G. | 2003 | Prospective randomized study on bilateral above-knee femoropopliteal revascularization: Polytetrafluoroethylene graft versus reversed saphenous vein | J Vasc Surg | 160 |
| **B56** | Ballotta, E. and Renon, L. and De Rossi, A. and Barbon, B. and Terranova, O. and Da Giau, G. | 2004 | Prospective randomized study on reversed saphenous vein infrapopliteal bypass to treat limb-threatening ischemia: common femoral artery versus superficial femoral or popliteal and tibial arteries as inflow | J Vasc Surg | 53 |
| **B57** | Goldman, M. and McCollum, C. | 1984 | A prospective randomized study to examine the effect of aspirin plus dipyridamole on the patency of prosthetic femoro-popliteal grafts | Vasc Surg | 136 |
| **B58** | Burger, D. H. and Kappetein, A. P. and Van Bockel, J. H. and Breslau, P. J. | 2000 | A prospective randomized trial comparing vein with polytetrafluoroethylene in above-knee femoropopliteal bypass grafting | J Vasc Surg | 93 |
| **B59** | Satiani, B. | 1985 | A prospective randomized trial of aspirin in femoral popliteal and tibial bypass grafts | Angiology | 122 |
| **B60** | Gupta, S. K. and Veith, F. J. and Kram, H. B. and Wengerter, K. R. | 1991 | Prospective, randomized comparison of ringed and nonringed polytetrafluoroethylene femoropopliteal bypass grafts: a preliminary report | J Vasc Surg | 168 |
| **B61** | Ozaki, C. K. and Hamdan, A. D. and Barshes, N. R. and Wyers, M. and Hevelone, N. D. and Belkin, M. and Nguyen, L. L. | 2015 | Prospective, randomized, multi-institutional clinical trial of a silver alginate dressing to reduce lower extremity vascular surgery wound complications | J Vasc Surg | 500 |
| **B62** | Harris, P. L. and How, T. V. and Jones, D. R. | 1987 | Prospectively randomized clinical trial to compare in situ and reversed saphenous vein grafts for femoropopliteal bypass | Br J Surg | 215 |
| **B63** | Abbott, W. M. and Green, R. M. and Matsumoto, T. and Wheeler, J. R. and Miller, N. and Veith, F. J. and Suggs, W. D. and Hollier, L. and Money, S. and Garrett, H. E. | 1997 | Prosthetic above-knee femoropopliteal bypass grafting: results of a multicenter randomized prospective trial. Above-Knee Femoropopliteal Study Group | J Vasc Surg | 244 |
| **B64** | Lepantalo, M. and Laurila, K. and Roth, W. D. and Rossi, P. and Lavonen, J. and Makinen, K. and Manninen, H. and Romsi, P. and Perala, J. and Bergqvist, D. and Scandinavian Thrupass Study, Group | 2009 | PTFE bypass or thrupass for superficial femoral artery occlusion? A randomised controlled trial | Eur J Vasc Endovasc Surg | 120 |
| **B65** | SCAMICOS | 2010 | PTFE bypass to below-knee arteries: distal vein collar or not? A prospective randomised multicentre study | Eur J Vasc Endovasc Surg | 352 |
| **B66** | McCollum, C. and Kenchington, G. and Alexander, C. and Franks, P. J. and Greenhalgh, R. M. | 1991 | PTFE or HUV for femoro-popliteal bypass: a multi-centre trial | Eur J Vasc Surg | 191 |
| **B67** | Aalders, G. J. and van Vroonhoven, T. J. and Lobach, H. J. and Wijffels, C. C. | 1988 | PTFE versus human umbilical vein in above knee femoro-popliteal bypass. Early results of a randomized clinical trial | J Cardiovasc Surg (Torino) | 96 |
| **B68** | Clyne, C. A. and Archer, T. J. and Atuhaire, L. K. and Chant, A. D. and Webster, J. H. | 1987 | Random control trial of a short course of aspirin and dipyridamole (Persantin) for femorodistal grafts | Br J Surg | 148 |
| **B69** | Eickhoff, J. H. and Buchardt Hansen, H. J. and Bromme, A. and Ericsson, B. F. and Kordt, K. F. and Mouritzen, C. and Myhre, H. O. and Norgren, L. and Rostad, H. and Trippestad, A. | 1983 | A randomized clinical trial of PTFE versus human umbilical vein for femoropopliteal bypass surgery. Preliminary results | Br J Surg | 104 |
| **B70** | Kedora, J. and Hohmann, S. and Garrett, W. and Munschaur, C. and Theune, B. and Gable, D. | 2007 | Randomized comparison of percutaneous Viabahn stent grafts vs prosthetic femoral-popliteal bypass in the treatment of superficial femoral arterial occlusive disease | J Vasc Surg | 86 |
| **B71** | Ouriel, K. and Veith, F. J. and Sasahara, A. A. | 1995 | Randomized comparison of thrombolysis and surgery. TOPAS Investigators. Thrombolysis or Peripheral Arterial Surgery | J Vasc Interv Radiol | 213 |
| **B72** | Lee, K. and Murphy, P. and Dubois, L. and Derose, G. and Forbes, T. and Power, A. | 2017 | Randomized control trial of negative pressure wound therarpy for high risk groin wounds in lower extremity revascularization | J Wound Care | 102 |
| **B73** | Tilanus, H. W. and Obertop, H. and Van Urk, H. | 1985 | Saphenous vein or PTFE for femoropopliteal bypass. A prospective randomized trial | Ann Surg | 49 |
| **B74** | Lindholt, J. S. and Gottschalksen, B. and Johannesen, N. and Dueholm, D. and Ravn, H. and Christensen, E. D. and Viddal, B. and FlÃ¸renes, T. and Pedersen, G. and Rasmussen, M. and Carstensen, M. and GrÃ¸ndal, N. and Fasting, H. | 2011 | The Scandinavian Propaten(Â®) trial - 1-year patency of PTFE vascular prostheses with heparin-bonded luminal surfaces compared to ordinary pure PTFE vascular prostheses - a randomised clinical controlled multi-centre trial | Eur J Vasc Endovasc Surg | 569 |
| **B75** | Gisbertz, S. S. and Ramzan, M. and Tutein Nolthenius, R. P. and van der Laan, L. and Overtoom, T. T. and Moll, F. L. and de Vries, J. P. | 2009 | Short-term results of a randomized trial comparing remote endarterectomy and supragenicular bypass surgery for long occlusions of the superficial femoral artery [the REVAS trial] | Eur J Vasc Endovasc Surg | 116 |
| **B76** | Veith, F. J. and Gupta, S. K. and Ascer, E. and White-Flores, S. and Samson, R. H. and Scher, L. A. and Towne, J. B. and Bernhard, V. M. and Bonier, P. and Flinn, W. R. and et al. | 1986 | Six-year prospective multicenter randomized comparison of autologous saphenous vein and expanded polytetrafluoroethylene grafts in infrainguinal arterial reconstructions | J Vasc Surg | 759 |
| **B77** | Wolterbeek, J. H. and van Leeuwen, A. A. and Breslau, P. J. | 2002 | Skin closure after infrainguinal bypass surgery: a prospective randomised study | Eur J Vasc Endovasc Surg | 170 |
| **B78** | Goueffic, Y. and Della Schiava, N. and Thaveau, F. and Rosset, E. and Favre, J. P. and Salomon du Mont, L. and Alsac, J. M. and Hassen-Khodja, R. and Reix, T. and Allaire, E. and Ducasse, E. and Soler, R. and Guyomarc'h, B. and Nasr, B. | 2017 | Stenting or Surgery for De Novo Common Femoral Artery Stenosis | JACC: Cardiovasc Interv | 117 |
| **B79** | Schulman, M. L. and Badhey, M. R. and Yatco, R. | 1987 | Superficial femoral-popliteal veins and reversed saphenous veins as primary femoropopliteal bypass grafts: a randomized comparative study | J Vasc Surg | 120 |
| **B80** | Vriens, B. H. and van Det, R. J. and Meerwaldt, R. and van der Palen, J. and Gerrits, D. G. and Zeebregts, C. J. and Geelkerken, R. H. | 2013 | Superior two-year results of externally unsupported polyester compared to supported grafts in above-knee bypass grafting: a multicenter randomised trial | Eur J Vasc Endovasc Surg | 265 |
| **B81** | Wolf, G. L. and Wilson, S. E. and Cross, A. P. and Deupree, R. H. and Stason, W. B. | 1993 | Surgery or balloon angioplasty for peripheral vascular disease: a randomized clinical trial. Principal investigators and their Associates of Veterans Administration Cooperative Study Number 199 | J Vasc Interv Radiol | 263 |
| **B82** | Linni, K. and Mader, N. and Aspalter, M. and Butturini, E. and Ugurluoglu, A. and Hitzl, W. and HÃ¶lzenbein, T. J. | 2012 | Ultrasonic vein mapping prior to infrainguinal autogenous bypass grafting reduces postoperative infections and readmissions | J Vasc Surg | 103 |
| **B83** | Ricco, J. B. | 1992 | Unilateral iliac artery occlusive disease: a randomized multicenter trial examining direct revascularization versus crossover bypass. Association Universitaire de Recherche en Chirurgie | Ann Vasc Surg | 143 |
| **B84** | Youssef, F. and Jenkins, M. P. and Dawson, K. J. and Berger, L. and Myint, F. and Hamilton, G. | 2005 | The value of suction wound drain after carotid and femoral artery surgery: a randomised trial using duplex assessment of the volume of post-operative haematoma | Eur J Vasc Endovasc Surg | 70 |
| **B85** | Sarac, T. P. and Huber, T. S. and Back, M. R. and Ozaki, C. K. and Carlton, L. M. and Flynn, T. C. and Seeger, J. M. | 1998 | Warfarin improves the outcome of infrainguinal vein bypass grafting at high risk for failure | J Vasc Surg | 56 |
| **B86** | Bosiers, M. and Setacci, C. and De Donato, G. and Torsello, G. and Silveira, P. G. and Deloose, K. and Scheinert, D. and Veroux, P. and Hendriks, J. and Maene, L. and Keirse, K. and Navarro, T. and Callaert, J. and Eckstein, H. H. and Teβarek, J. and Giaquinta, A. and Wauters, J. | 2020 | ZILVERPASS Study: ZILVER PTX Stent vs Bypass Surgery in Femoropopliteal Lesions | JEVT | 220 |
| **B87** | The STILE Investigators | 1994 | Results of a Prospective Randomized Trial Evaluating Surgery Versus Thrombolysis for Ischemia of the Lower Extremity: The STILE Trial | Annals of surgery | 393 |
| **B88** | Edward O. McFalls, M.D., Ph.D., Herbert B. Ward, M.D., Ph.D., Thomas E. Moritz, M.S., Steven Goldman, M.D., William C. Krupski, M.D.,* Fred Littooy, M.D., Gordon Pierpont, M.D., Steve Santilli, M.D., Joseph Rapp, M.D., Brack Hattler, M.D., Kendrick Shunk, M.D., Ph.D., Connie Jaenicke, R.N., B.S.N., Lizy Thottapurathu, M.S., Nancy Ellis, M.S., Domenic J. Reda, Ph.D., and William G. Henderson, Ph.D. | 2004 | Coronary-Artery Revascularization before Elective Major Vascular Surgery | N Engl J Med | 510 |
| **B89** | A. Farber, M.T. Menard, M.S. Conte, J.A. Kaufman, R.J. Powell, N.K. Choudhry, T.H. Hamza, S.F. Assmann,* M.A. Creager, M.J. Cziraky, M.D. Dake, M.R. Jaff, D. Reid, F.S. Siami, G. Sopko, C.J. White, M. van Over, M.B. Strong, M.F. Villarreal, M. McKean, E. Azene, A. Azarbal, A. Barleben, D.K. Chew, L.C. Clavijo, Y. Douville, L. Findeiss, N. Garg, W. Gasper, K.A. Giles, P.P. Goodney, B.M. Hawkins, C.R. Herman, J.A. Kalish, M.C. Koopmann, I.A. Laskowski, C. Mena‑Hurtado, R. Motaganahalli, V.L. Rowe, A. Schanzer, P.A. Schneider, J.J. Siracuse, M. Venermo, and K. Rosenfield, for the BEST-CLI Investigators | 2022 | Surgery or Endovascular Therapy for Chronic Limb-Threatening Ischemia | N Engl J Med | 1830 |
| **B90** | Gussoni, G. and De Donato, G. and Andreozzi, G. M. and Bonizzoni, E. and Mazzone, A. and Odero, A. and Paroni, G. and Setacci, C. and Settembrini, P. and Veglia, F. and Martini, R. and Setacci, F. and Palombo, D. | 2006 | The ILAILL study: Iloprost as adjuvant to surgery for acute ischemia of lower limbs - A randomized, placebo-controlled, double-blind study by the Italian Society for Vascular and Endovascular Surgery | Ann Surg | 300 |
| **B91** | Li, J. and Wang, B. and Wang, Y. and Wu, F. and Li, P. and Li, Y. and Zhao, L. and Cui, W. and Ding, Y. and An, Q. and Si, J. | 2013 | Therapeutic effect of liposomal prostaglandin E1 in acute lower limb ischemia as an adjuvant to hybrid procedures | Exp Ther Med | 204 |
| **B92** | Marc P. Bonaca, M.D., M.P.H., Rupert M. Bauersachs, M.D., Sonia S. Anand, M.D., E. Sebastian Debus, M.D., Ph.D., Mark R. Nehler, M.D., Manesh R. Patel, M.D., Fabrizio Fanelli, M.D., Warren H. Capell, M.D., Lihong Diao, M.D., Nicole Jaeger, M.S., Connie N. Hess, M.D., M.H.S., Akos F. Pap, M.Sc., John M. Kittelson, Ph.D., Ivan Gudz, M.D., Ph.D., Lajos Mátyás, M.D., Dainis K. Krievins, M.D., Rafael Diaz, M.D., Marianne Brodmann, M.D., Eva Muehlhofer, M.D., Lloyd P. Haskell, M.D., Scott D. Berkowitz, M.D., and William R. Hiatt, M.D. | 2020 | Rivaroxaban in Peripheral Artery Disease after Revascularization (VOYAGER PAD) | N Engl J Med | 6564 |
| **B93** | Bradbury, A. W. and Moakes, C. A. and Popplewell, M. and Meecham, L. and Bate, G. R. and Kelly, L. and Chetter, I. and Diamantopoulos, A. and Ganeshan, A. and Hall, J. and Hobbs, S. and Houlind, K. and Jarrett, H. and Lockyer, S. and Malmstedt, J. and Patel, J. V. and Patel, S. and Rashid, S. T. and Saratzis, A. and Slinn, G. and Scott, D. J. A. and Zayed, H. and Deeks, J. J. and BASIL-2 Investigators | 2023 | A vein bypass first versus a best endovascular treatment first revascularisation strategy for patients with chronic limb threatening ischaemia who required an infra-popliteal, with or without an additional more proximal infra-inguinal revascularisation procedure to restore limb perfusion (BASIL-2): an open-label, randomised, multicentre, phase 3 trial | Lancet | 345 |
| **B94** | Enzmann, F. K. and Nierlich, P. and Hölzenbein, T. and Aspalter, M. and Kluckner, M. and Hitzl, W. and Opperer, M. and Linni, K. | 2023 | Vein bypass versus nitinol stent in long femoropopliteal lesions: 4-year results of a randomized controlled trial | Ann Surg | 209 |

| **Carotid** | | | | | |
| --- | --- | --- | --- | --- | --- |
| **Study identifier** | **Authors** | **Year** | **Title** | **Journal** | **Number of trial participants** |
| **C1** | Space Collaborative Group and Ringleb, P. A. and Allenberg, J. and Bruckmann, H. and Eckstein, H. H. and Fraedrich, G. and Hartmann, M. and Hennerici, M. and Jansen, O. and Klein, G. and Kunze, A. and Marx, P. and Niederkorn, K. and Schmiedt, W. and Solymosi, L. and Stingele, R. and Zeumer, H. and Hacke, W. | 2006 | 30 day results from the SPACE trial of stent-protected angioplasty versus carotid endarterectomy in symptomatic patients: a randomised non-inferiority trial | Lancet | 1200 |
| **C2** | Sindjelic, R. P. and Vlajkovic, G. P. and Davidovic, L. B. and Markovic, D. Z. and Markovic, M. D. | 2010 | The addition of fentanyl to local anesthetics affects the quality and duration of cervical plexus block: A randomized, controlled trial | Anesth Analg | 77 |
| **C3** | Madro, P. and Dabrowska, A. and Jarecki, J. and Garba, P. | 2016 | Anaesthesia for carotid endarterectomy. Ultrasound-guided superficial/intermediate cervical plexus block combined with carotid sheath infiltration | Anestezjol Intens Ter | 98 |
| **C4** | Godet, G. and Reina, M. and Raux, M. and Amour, J. and De Castro, V. and Coriat, P. | 2004 | Anaesthesia for carotid endarterectomy: comparison of hypnotic- and opioid-based techniques | Br J Anaesth | 46 |
| **C5** | Pratschner, T. and Kretschmer, G. and Prager, M. and Wenzl, E. and Polterauer, P. and Ehringer, H. and Horvath, R. and Holzner, H. | 1990 | Antiplatelet therapy following carotid bifurcation endarterectomy. Evaluation of a controlled clinical trial. Prognostic significance of histologic plaque examination of behalf of survival | Eur J Vasc Endovasc Surg | 66 |
| **C6** | Amato, B. and Compagna, R. and Amato, M. and Gallelli, L. and de Franciscis, S. and Serra, R. | 2015 | Aterofisiol in carotid plaque evolution | Drug Des Devel Ther | 214 |
| **C7** | Cortellaro, M. and Confrancesco, E. and Arbustini, E. and Rossi, F. and Negri, A. and Tremoli, E. and Gabrielli, L. and Camera, M. | 2002 | Atorvastatin and thrombogenicity of the carotid atherosclerotic plaque: The ATROCAP Study | Thromb Haemost | 59 |
| **C8** | Puato, M. and Faggin, E. and Rattazzi, M. and Zambon, A. and Cipollone, F. and Grego, F. and Ganassin, L. and Plebani, M. and Mezzetti, A. and Pauletto, P. | 2010 | Atorvastatin reduces macrophage accumulation in atherosclerotic plaques: a comparison of a nonstatin-based regimen in patients undergoing carotid endarterectomy | Stroke | 60 |
| **C9** | Payne, D. A. and Jones, C. I. and Hayes, P. D. and Thompson, M. M. and London, N. J. and Bell, P. R. and Goodall, A. H. and Naylor, A. R. | 2004 | Beneficial effects of clopidogrel combined with aspirin in reducing cerebral emboli in patients undergoing carotid endarterectomy | Circulation | 100 |
| **C10** | Barnett, H. J. and Taylor, D. W. and Eliasziw, M. and Fox, A. J. and Ferguson, G. G. and Haynes, R. B. and Rankin, R. N. and Clagett, G. P. and Hachinski, V. C. and Sackett, D. L. and Thorpe, K. E. and Meldrum, H. E. and Spence, J. D. | 1998 | Benefit of carotid endarterectomy in patients with symptomatic moderate or severe stenosis. North American Symptomatic Carotid Endarterectomy Trial Collaborators | N Engl J Med | 2267 |
| **C11** | Marien, B. J. and Raffetto, J. D. and Seidman, C. S. and LaMorte, W. W. and Menzoian, J. O. | 2002 | Bovine pericardium vs dacron for patch angioplasty after carotid endarterectomy: a prospective randomized study | Arch Surg | 92 |
| **C12** | Tauber, H. and Streif, W. and Gebetsberger, J. and Gasteiger, L. and Pierer, E. and Knoflach, M. and Fraedrich, G. and Gummerer, M. and Fritz, J. and Velik-Salchner, C. | 2021 | Cardiac output and cerebral blood flow during carotid surgery in regional versus general anesthesia: A prospective randomized controlled study | J Vasc Surg | 90 |
| **C13** | Marietta, D. R. and Lunn, J. K. and Ruby, E. I. and Hill, G. E. | 1998 | Cardiovascular stability during carotid endarterectomy: endotracheal intubation versus laryngeal mask airway | J Clin Anesth | 61 |
| **C14** | Brooks, W. H. and McClure, R. R. and Jones, M. R. and Coleman, T. L. and Breathitt, L. and Solomon, R. A. and Fiorella, D. and Albuquerque, F. C. and Rosenwasser, R. H. and Cullen, S. and Higashida, R. T. and Harrigan, M. R. and Hopkins, L. N. | 2004 | Carotid Angioplasty and Stenting versus Carotid Endarterectomy for Treatment of Asymptomatic Carotid Stenosis: A Randomized Trial in a Community Hospital | Neurosurgery | 85 |
| **C15** | Brooks, W. H. and McClure, R. R. and Jones, M. R. and Coleman, T. C. and Breathitt, L. | 2001 | Carotid angioplasty and stenting versus carotid endarterectomy: randomized trial in a community hospital | J Am Coll Cardiol | 104 |
| **C16** | Featherstone, R. L. and Dobson, J. and Ederle, J. and Doig, D. and Bonati, L. H. and Morris, S. and Patel, N. V. and Brown, M. M. | 2016 | Carotid artery stenting compared with endarterectomy in patients with symptomatic carotid stenosis (International Carotid Stenting Study): a randomised controlled trial with cost-effectiveness analysis | Health Technol Assess | 1713 |
| **C17** | Shaw, D. A. and Venables, G. S. and Cartlidge, N. E. F. | 1984 | Carotid endarterectomy in patients with transient cerebral ischaemia | J Neurol Sci | 41 |
| **C18** | Mannheim, D. and Weller, B. and Vahadim, E. and Karmeli, R. | 2005 | Carotid endarterectomy with a polyurethane patch versus primary closure: a prospective randomized study | J Vasc Surg | 404 |
| **C19** | Ignatenko, P. and Novikova, O. and Gostev, A. and Starodubtsev, V. and Zeidlits, G. and Kuznetsov, K. and Starodubtseva, A. and Karpenko, A. | 2019 | Carotid Endarterectomy with Autoarterial Remodeling of Bifurcation of the Common Carotid Artery and Carotid Endarterectomy with Patch Closure: Comparison of Methods | J Stroke Cerebrovasc Dis | 200 |
| **C20** | Ballotta, E. and Da Giau, G. and Saladini, M. and Abbruzzese, E. and Renon, L. and Toniato, A. | 1999 | Carotid endarterectomy with patch closure versus carotid eversion endarterectomy and reimplantation: a prospective randomized study | Surgery | 310 |
| **C21** | Deriu, G. and Milite, D. and Damiani, N. and Mercurio, D. and Bonvicini, C. and Lepidi, S. and Grego, F. | 2000 | Carotid endarterectomy without angiography: A prospective randomised pilot study | Eur J Vasc Endovasc Surg | 186 |
| **C22** | Mantese, V. A. and Timaran, C. H. and Chiu, D. and Begg, R. J. and Brott, T. G. and for the CREST Investigators | 2010 | Carotid revascularization endarterectomy versus stenting trial (CREST) | Vascular | 2502 |
| **C23** | CaRESS Steering Committee | 2005 | Carotid Revascularization Using Endarterectomy or Stenting Systems (CaRESS) phase I clinical trial: 1-year results | J Vasc Surg | 397 |
| **C24** | Simka, M. and Bryll, A. and Piwowarczyk, M. and Gajos, G. and Popiela, T. and Latacz, P. | 2019 | Cerebral ischemic lesions on diffusionweighted magnetic resonance imaging after carotid eversion endarterectomy vs carotid stenting with a proximal protection device: Results of a randomized prospective trial | Pol Arch Med Wewn | 31 |
| **C25** | Leoni, A. and Magrin, S. and Mascotto, G. and Rigamonti, A. and Gallioli, G. and Muzzolon, F. and Fanelli, G. and Casati, A. | 2000 | Cervical plexus anesthesia for carotid endarterectomy: comparison of ropivacaine and mepivacaine | Can J Anaesth | 60 |
| **C26** | Melissano, G. and Blasi, F. and Esposito, G. and Tarsia, P. and Dordoni, L. and Arosio, C. and Tshomba, Y. and Fagetti, L. and Allegra, L. and Chiesa, R. | 1999 | Chlamydia pneumoniae eradication from carotid plaques. Results of an open, randomised treatment study | Eur J Vasc Endovasc Surg | 32 |
| **C27** | Schneemilch, C. E. and Bachmann, H. and Ulrich, A. and Elwert, R. and Halloul, Z. and Hachenberg, T. | 2006 | Clonidine decreases stress response in patients undergoing carotid endarterectomy under regional anesthesia: a prospective, randomized, double-blinded, placebo-controlled study | Anesth Analg | 80 |
| **C28** | Kalimeris, K. and Kouni, S. and Kostopanagiotou, G. and Nomikos, T. and Fragopoulou, E. and Kakisis, J. and Vasdekis, S. and Matsota, P. and Pandazi, A. | 2013 | Cognitive function and oxidative stress after carotid endarterectomy: comparison of propofol to sevoflurane anesthesia | J Cardiothorac Vasc Anesth | 50 |
| **C29** | McGlade, D. P. and Murphy, P. M. and Davies, M. J. and Scott, D. A. and Silbert, B. S. | 1996 | Comparative effects of plain and epinephrine-containing bupivacaine on the hemodynamic response to cervical plexus anesthesia in patients undergoing carotid endarterectomy | J Cardiothorac Vasc Anesth | 40 |
| **C30** | Aliev, V. A. and Yavorovskii, A. G. and Shaposhnikov, A. A. and Loriya, I. Z. and Vetsheva, M. S. | 2019 | Comparative evaluation of modern inhalation anesthetics in carotid endarterectomy | Obs Reanimatol | 67 |
| **C31** | Pinto Neto, W. and Issy, A. M. and Sakata, R. K. | 2009 | A comparative study between bupivacaine and clonidine associated with bupivacaine in cervical plexus block for carotid endarterectomy. [Portuguese, English] | Rev Bras Anestesiol | 30 |
| **C32** | Molnar, R. R. and Davies, M. J. and Scott, D. A. and Silbert, B. S. and Mooney, P. H. | 1997 | Comparison of clonidine and epinephrine in lidocaine for cervical plexus block | Reg Anesth | 40 |
| **C33** | Kavakli, A. S. and Kavrut Ozturk, N. and Umut Ayotlu, R. and Satdic, K. and Cakmak, G. and Inanotlu, K. and Emmiler, M. | 2016 | Comparison of combined (deep and superficial) and intermediate cervical plexus block by use of ultrasound guidance for carotid endarterectomy | J Cardiothorac Vasc Anesth | 48 |
| **C34** | McCutcheon, C. A. and Orme, R. M. and Scott, D. A. and Davies, M. J. and McGlade, D. P. | 2006 | A comparison of dexmedetomidine versus conventional therapy for sedation and hemodynamic control during carotid endarterectomy performed under regional anesthesia | Anesth Analg | 56 |
| **C35** | Kougias, P. and Collins, R. and Pastorek, N. and Sharath, S. and Barshes, N. R. and McCulloch, K. and Pisimisis, G. and Berger, D. H. | 2015 | Comparison of domain-specific cognitive function after carotid endarterectomy and stenting | J Vasc Surg | 60 |
| **C36** | Ramachandran, S. K. and Picton, P. and Shanks, A. and Dorje, P. and Pandit, J. J. | 2011 | Comparison of intermediate vs subcutaneous cervical plexus block for carotid endarterectomy | Br J Anaesth | 46 |
| **C37** | Crawley, F. and Stygall, J. and Lunn, S. and Harrison, M. and Brown, M. M. and Newman, S. | 2000 | Comparison of microembolism detected by transcranial Doppler and neuropsychological sequelae of carotid surgery and percutaneous transluminal angioplasty | Stroke | 50 |
| **C38** | Doyle, P. W. and Coles, J. P. and Leary, T. M. and Brazier, P. and Gupta, A. K. | 2001 | A comparison of remifentanil and fentanyl in patients undergoing carotid endarterectomy | Eur J Anaesthesiol | 33 |
| **C39** | Godet, G. and Watremez, C. and El Kettani, C. and Soriano, C. and Coriat, P. | 2001 | A comparison of sevoflurane, target-controlled infusion propofol, and propofol/isoflurane anesthesia in patients undergoing carotid surgery: a quality of anesthesia and recovery profile | Anesth Analg | 45 |
| **C40** | Pandit, J. J. and Bree, S. and Dillon, P. and Elcock, D. and McLaren, I. D. and Crider, B. | 2000 | A comparison of superficial versus combined (superficial and deep) cervical plexus block for carotid endarterectomy: a prospective, randomized study | Anesth Analg | 40 |
| **C41** | Park, S. and Yook, K. and Yoo, K. Y. and Choi, J. I. and Bae, H. B. and You, Y. and Jin, B. and Jeong, S. | 2019 | Comparison of the effect of sevoflurane or propofol anesthesia on the regional cerebral oxygen saturation in patients undergoing carotid endarterectomy: a prospective, randomized controlled study | BMC Anesthesiol | 74 |
| **C42** | Mouren, S. and De Winter, G. and Guerrero, S. P. and Baillard, C. and Bertrand, M. and Coriat, P. | 2001 | The continuous recording of blood pressure in patients undergoing carotid surgery under remifentanil versus sufentanil analgesia | Anesth Analg | 56 |
| **C43** | Kazimierczak, A. and Rybicka, A. and Rynio, P. and Gutowski, P. and Wiernicki, I. | 2018 | Cosmetic effects of skin-crease camouflage incision versus longitudinal incision following carotid endarterectomy | Wideochir Inne Tech Maloinwazyjne | 200 |
| **C44** | Bekker, A. Y. and Basile, J. and Gold, M. and Riles, T. and Adelman, M. and Cuff, G. and Mathew, J. P. and Goldberg, J. D. | 2004 | Dexmedetomidine for awake carotid endarterectomy: efficacy, hemodynamic profile, and side effects | J Neurosurg Anesthesiol | 66 |
| **C45** | Ge, Y. and Li, Q. and Nie, Y. and Gao, J. and Luo, K. and Fang, X. and Wang, C. | 2019 | Dexmedetomidine improves cognition after carotid endarterectomy by inhibiting cerebral inflammation and enhancing brain-derived neurotrophic factor expression | J Int Med Res | 49 |
| **C46** | Vanmaele, R. G. and Van Schil, P. E. and DeMaeseneer, M. G. and Meese, G. and Lehert, P. and Van Look, R. F. | 1994 | Division-endarterectomy-anastomosis of the internal carotid artery: a prospective randomized comparative study | Cardiovasc Surg | 200 |
| **C47** | Smith, J. S. and Roizen, M. F. and Cahalan, M. K. and Benefiel, D. J. and Beaupre, P. N. and Sohn, Y. J. and Byrd, B. F. and Schiller, N. B. and Stoney, R. J. and Ehrenfeld, W. K. and Ellis, J. E. and Aronson, S. | 1988 | Does anesthetic technique make a difference? Augmentation of systolic blood pressure during carotid endarterectomy: Effects of phenylephrine versus light anesthesia and of isoflurance versus halothane on the incidence of myocardial ischemia | Anesthesiology | 60 |
| **C48** | Welsh, S. and Mead, G. and Chant, H. and Picton, A. and O'Neill, P. A. and McCollum, C. N. | 2004 | Early carotid surgery in acute stroke: a multicentre randomised pilot study | Cerebrovasc Dis | 40 |
| **C49** | Ballotta, E. and Da Giau, G. and Baracchini, C. and Abbruzzese, E. and Saladini, M. and Meneghetti, G. | 2002 | Early versus delayed carotid endarterectomy after a nondisabling ischemic stroke: A prospective randomized study | Surgery | 86 |
| **C50** | McKevitt, F. M. and Sivaguru, A. and Venables, G. S. and Cleveland, T. J. and Gaines, P. A. and Beard, J. D. and Channer, K. S. | 2003 | Effect of treatment of carotid artery stenosis on blood pressure: a comparison of hemodynamic disturbances after carotid endarterectomy and endovascular treatment | Stroke | 103 |
| **C51** | Kavakli, A. S. and Kavrut Ozturk, N. and Yavuzel Adas, H. and Kudsioglu, S. T. and Ayoglu, R. U. and Ozmen, S. and Sagdic, K. and Yapici, N. | 2019 | The effects of music on anxiety and pain in patients during carotid endarterectomy under regional anesthesia: A randomized controlled trial | Complement Ther Med | 70 |
| **C52** | Rossel, T. and Uhlig, C. and Pietsch, J. and Ludwig, S. and Koch, T. and Richter, T. and Spieth, P. M. and Kersting, S. | 2019 | Effects of regional anesthesia techniques on local anesthetic plasma levels and complications in carotid surgery: a randomized controlled pilot trial | BMC Anesthesiol | 30 |
| **C53** | Hobson, R. W., 2nd and Weiss, D. G. and Fields, W. S. and Goldstone, J. and Moore, W. S. and Towne, J. B. and Wright, C. B. | 1993 | Efficacy of carotid endarterectomy for asymptomatic carotid stenosis. The Veterans Affairs Cooperative Study Group | N Engl J Med | 444 |
| **C54** | Cawood, A. L. and Ding, R. and Napper, F. L. and Young, R. H. and Williams, J. A. and Ward, M. J. and Gudmundsen, O. and Vige, R. and Payne, S. P. and Ye, S. and Shearman, C. P. and Gallagher, P. J. and Grimble, R. F. and Calder, P. C. | 2010 | Eicosapentaenoic acid (EPA) from highly concentrated n-3 fatty acid ethyl esters is incorporated into advanced atherosclerotic plaques and higher plaque EPA is associated with decreased plaque inflammation and increased stability | Atherosclerosis | 121 |
| **C55** | Tytgat, Shaj and Laman, D. M. and Rijken, A. M. and Klicks, R. and Voorwinde, A. and Ultee, J. M. and Van Duijn, H. | 2005 | Emboli rate during and early after carotid endarterectomy after a single preoperative dose of 120 mg acetylsalicylic acid - A prospective double-blind placebo controlled randomised trial | Eur J Vasc Endovasc Surg | 100 |
| **C56** | Mas, J. L. and Chatellier, G. and Beyssen, B. and Branchereau, A. and Moulin, T. and Becquemin, J. P. and Larrue, V. and Lievre, M. and Leys, D. and Bonneville, J. F. and Watelet, J. and Pruvo, J. P. and Albucher, J. F. and Viguier, A. and Piquet, P. and Garnier, P. and Viader, F. and Touze, E. and Giroud, M. and Hosseini, H. and Pillet, J. C. and Favrole, P. and Neau, J. P. and Ducrocq, X. and Investigators, Eva- S. | 2006 | Endarterectomy versus stenting in patients with symptomatic severe carotid stenosis | N Engl J Med | 527 |
| **C57** | CAVATAS investigators | 2001 | Endovascular versus surgical treatment in patients with carotid stenosis in the Carotid and Vertebral Artery Transluminal Angioplasty Study (CAVATAS): a randomised trial | Lancet | 504 |
| **C58** | Zogogiannis, I. D. and Iatrou, C. A. and Lazarides, M. K. and Vogiatzaki, T. D. and Wachtel, M. S. and Chatzigakis, P. K. and Dimitriou, V. K. | 2011 | Evaluation of an intraoperative algorithm based on near-infrared refracted spectroscopy monitoring, in the intraoperative decision for shunt placement, in patients undergoing carotid endarterectomy | Middle East J Anaesthesiol | 253 |
| **C59** | Grego, F. and Milite, D. and Cognolato, D. and Frigatti, P. and Morelli, I. and Bonvini, S. and Damiani, N. and Deriu, G. P. | 2001 | External jugular vein patch versus PTFE patch after carotid endarterectomy: A randomised prospective study | G Ital Chir Vasc | 160 |
| **C60** | Harker, L. A. and Bernstein, E. F. and Dilley, R. B. and Scala, T. E. and Sise, M. J. and Hye, R. J. and Otis, S. M. and Roberts, R. S. and Gent, M. | 1992 | Failure of aspirin plus dipyridamole to prevent restenosis after carotid endarterectomy | Ann Intern Med | 163 |
| **C61** | Gough, M. J. and Bodenham, A. and Horrocks, M. and Colam, B. and Lewis, S. C. and Rothwell, P. M. and Banning, A. P. and Torgerson, D. and Gough, M. and Dellagrammaticas, D. and Leigh-Brown, A. and Liapis, C. and Warlow, C. | 2008 | GALA: An international multicentre randomised trial comparing general anaesthesia versus local anaesthesia for carotid surgery | Trials | 3526 |
| **C62** | Jellish, W. S. and Sheikh, T. and Baker, W. H. and Louie, E. K. and Slogoff, S. | 2003 | Hemodynamic stability, myocardial ischemia, and perioperative outcome after carotid surgery with remifentanil/propofol or isoflurane/fentanyl anesthesia | J Neurosurg Anesthesiol | 60 |
| **C63** | Chiu, C. and Heyer, E. J. and Rampersad, A. D. and Zurica, J. and Ornstein, E. and Sahlein, D. H. and Sciacca, R. R. and Connolly, E. S., Jr. | 2006 | High dose magnesium infusions are not associated with increased pressor requirements after carotid endarterectomy | Neurosurgery | 80 |
| **C64** | Petrucci, E. and Cofini, V. and Pizzi, B. and Coletta, R. and Blasetti, A. G. and Necozione, S. and Fusco, P. and Marinangeli, F. | 2020 | Intermediate Cervical Plexus Block in the Management of Persistent Postoperative Pain Post Carotid Endarterectomy: A Prospective, Randomized, Controlled, Clinical Trial | Pain Physician | 98 |
| **C65** | Nasr, H. and Torsney, E. and Poston, R. N. and Hayes, L. and Gaze, D. C. and Basser, R. and Thompson, M. M. and Loftus, I. M. and Cockerill, G. W. | 2015 | Investigating the Effect of a Single Infusion of Reconstituted High-Density Lipoprotein in Patients with Symptomatic Carotid Plaques | Ann Vasc Surg | 40 |
| **C66** | Hussien, G. Z. and Elbadawy, A. M. and Elshamaa, H. A. | 2017 | Lactate/pyruvate monitoring during carotid endarterectomy under general anaesthesia versus cervical plexus block: A randomised controlled study | Indian J Anaesth | 36 |
| **C67** | Yusof, H. M. and Cawood, A. L. and Ding, R. and Williams, J. A. and Napper, F. L. and Shearman, C. P. and Grimble, R. F. and Payne, S. P. K. and Calder, P. C. | 2013 | Limited impact of 2 g/day Omega-3 fatty acid ethyl esters (Omacor) on plasma lipids and inflammatory markers in patients awaiting carotid endarterectomy | Mar Drugs | 121 |
| **C68** | Cherprenet, A. L. and Rambourdin-Perraud, M. and Laforet, S. and Faure, M. and Guesmi, N. and Baud, C. and Rosset, E. and Schoeffler, P. and Duale, C. | 2015 | Local anaesthetic infiltration at the end of carotid endarterectomy improves post-operative analgesia | Acta Anaesthesiol Scand | 46 |
| **C69** | Sbarigia, E. and DarioVizza, C. and Antonini, M. and Speziale, F. and Maritti, M. and Fiorani, B. and Fedele, F. and Fiorani, P. | 1999 | Locoregional versus general anesthesia in carotid surgery: is there an impact on perioperative myocardial ischemia? Results of a prospective monocentric randomized trial | J Vasc Surg | 107 |
| **C70** | Katz, D. and Snyder, S. O. and Gandhi, R. H. and Wheeler, J. R. and Gregory, R. T. and Gayle, R. G. and Parent, F. N., 3rd | 1994 | Long-term follow-up for recurrent stenosis: a prospective randomized study of expanded polytetrafluoroethylene patch angioplasty versus primary closure after carotid endarterectomy | J Vasc Surg | 87 |
| **C71** | McMahon, G. S. and Webster, S. E. and Hayes, P. D. and Jones, C. I. and Goodall, A. H. and Naylor, A. R. | 2009 | Low Molecular Weight Heparin Significantly Reduces Embolisation After Carotid Endarterectomy - A Randomised Controlled Trial | Eur J Vasc Endovasc Surg | 183 |
| **C72** | Pandazi, A. and Karamanis, P. and Sidiropoulou, T. and Matsota, P. and Papasideris, C. and Niokou, D. and Kostopanagiotou, G. | 2011 | Low-dose (1 microg/kg) clonidine premedication and hypotension after carotid artery surgery | Vasc Endovascular Surg | 84 |
| **C73** | Taylor, D. W. and Barnett, H. J. M. and Haynes, R. B. and Ferguson, G. G. and Sackett, D. L. and Thorpe, K. E. and Simard, D. and Silver, F. L. and Hachinski, V. and Clagett, G. P. and Barnes, R. and Spence, J. D. | 1999 | Low-dose and high-dose acetylsalicylic acid for patients undergoing carotid endarterectomy: A randomised controlled trial | Lancet | 2849 |
| **C74** | Tsujikawa, S. and Ikeshita, K. | 2019 | Low-dose dexmedetomidine provides hemodynamics stabilization during emergence and recovery from general anesthesia in patients undergoing carotid endarterectomy: a randomized double-blind, placebo-controlled trial | J Anesth | 50 |
| **C75** | Kolos, I. and Troitskiy, A. and Balakhonova, T. and Shariya, M. and Skrypnik, D. and Tvorogova, T. and Deev, A. and Boytsov, S. | 2015 | Modern medical treatment with or without carotid endarterectomy for severe asymptomatic carotid atherosclerosis | J Vasc Surg | 55 |
| **C76** | Dorman, T. and Thompson, D. A. and Breslow, M. J. and Lipsett, P. A. and Rosenfeld, B. A. | 2001 | Nicardipine versus nitroprusside for breakthrough hypertension following carotid endarterectomy | J Clin Anesth | 60 |
| **C77** | Badner, N. H. and Beattie, W. S. and Freeman, D. and Spence, J. D. | 2000 | Nitrous oxide-induced increased homocysteine concentrations are associated with increased postoperative myocardial ischemia in patients undergoing carotid endarterectomy | Anesth Analg | 90 |
| **C78** | McCarthy, R. J. and Nasr, M. K. and McAteer, P. and Horrocks, M. | 2002 | Physiological advantages of cerebral blood flow during carotid endarterectomy under local anaesthesia. A randomised clinical trial | Eur J Vasc Endovasc Surg | 67 |
| **C79** | Yang, C. L. and Tan, Y. H. and Jiang, X. X. and Meng, F. Y. and Wu, Y. L. and Chen, Q. L. and Ma, L. L. and Wang, L. X. | 2012 | Pre-operative education and counselling are associated with reduced anxiety symptoms following carotid endarterectomy: a randomized and open-label study | Eur J Cardiovasc Nurs | 120 |
| **C80** | Davies, M. J. and Dysart, R. H. and Silbert, B. S. and Scott, D. A. and Cook, R. J. | 1992 | Prevention of tachycardia with atenolol pretreatment for carotid endarterectomy under cervical plexus blockade | Anaesth Intensive Care | 40 |
| **C81** | Meerwaldt, R. and Lansink, K. W. and Blomme, A. M. and Fritschy, W. M. | 2008 | Prospective randomized study of carotid endarterectomy with Fluoropassiv thin wall carotid patch versus venous patch | Eur J Vasc Endovasc Surg | 87 |
| **C82** | AbuRahma, A. F. and Hannay, R. S. and Khan, J. H. and Robinson, P. A. and Hudson, J. K. and Davis, E. A. | 2002 | Prospective randomized study of carotid endarterectomy with polytetrafluoroethylene versus collagen-impregnated Dacron (Hemashield) patching: perioperative (30-day) results | J Vasc Surg | 200 |
| **C83** | Ballotta, E. and Renon, L. and Da Giau, G. and Barbon, B. and De Rossi, A. and Baracchini, C. | 2005 | Prospective randomized study on asymptomatic severe carotid stenosis and perioperative stroke risk in patients undergoing major vascular surgery: prophylactic or deferred carotid endarterectomy? | Ann Vasc Surg | 79 |
| **C84** | Mannheim, D. and Karmeli, R. | 2017 | A prospective randomized trial comparing endarterectomy to stenting in severe asymptomatic carotid stenosis | J Cardiovasc Surg (Torino) | 136 |
| **C85** | AbuRahma, A. F. and Stone, P. A. and Flaherty, S. K. and AbuRahma, Z. | 2007 | Prospective randomized trial of ACUSEAL (Gore-Tex) versus Hemashield-Finesse patching during carotid endarterectomy: early results | J Vasc Surg | 200 |
| **C86** | AbuRahma, A. F. and Robinson, P. A. and Saiedy, S. and Richmond, B. K. and Khan, J. | 1999 | Prospective randomized trial of bilateral carotid endarterectomies: Primary closure versus patching | Stroke | 74 |
| **C87** | Aburahma, A. F. and Stone, P. A. and Hass, S. M. and Dean, L. S. and Habib, J. and Keiffer, T. and Emmett, M. | 2010 | Prospective randomized trial of routine versus selective shunting in carotid endarterectomy based on stump pressure | J Vasc Surg | 200 |
| **C88** | Stoneham, M. D. and Doyle, A. R. and Knighton, J. D. and Dorje, P. and Stanley, J. C. | 1998 | Prospective, randomized comparison of deep or superficial cervical plexus block for carotid endarterectomy surgery | Anesthesiology | 40 |
| **C89** | O'Hara, P. J. and Hertzer, N. R. and Mascha, E. J. and Krajewski, L. P. and Clair, D. G. and Ouriel, K. | 2002 | A prospective, randomized study of saphenous vein patching versus synthetic patching during carotid endarterectomy | J Vasc Surg | 195 |
| **C90** | Axisa, B. and Loftus, I. M. and Naylor, A. R. and Goodall, S. and Jones, L. and Bell, P. R. and Thompson, M. M. | 2002 | Prospective, randomized, double-blind trial investigating the effect of doxycycline on matrix metalloproteinase expression within atherosclerotic carotid plaques | Stroke | 100 |
| **C91** | Yadav, J. S. and Wholey, M. H. and Kuntz, R. E. and Fayad, P. and Katzen, B. T. and Mishkel, G. J. and Bajwa, T. K. and Whitlow, P. and Strickman, N. E. and Jaff, M. R. and Popma, J. J. and Snead, D. B. and Cutlip, D. E. and Firth, B. G. and Ouriel, K. and Stenting and Angioplasty with Protection in Patients at High Risk for Endarterectomy, Investigators | 2004 | Protected carotid-artery stenting versus endarterectomy in high-risk patients | N Engl J Med | 334 |
| **C92** | Wang, Q. and Li, Y. H. and Wang, T. L. and Feng, H. and Cai, B. | 2015 | Protective Effect of Low-dose Sevoflurane Inhalation and Propofol Anesthesia on the Myocardium after Carotid Endarterectomy: A Randomized Controlled Trial | Chin Med J (Engl) | 122 |
| **C93** | Kuliha, M. and Roubec, M. and ProchÃ¡zka, V. and Jonszta, T. and HrbÃ¡Ä, T. and Havelka, J. and GoldÃ­rovÃ¡, A. and LangovÃ¡, K. and Herzig, R. and Å koloudÃ­k, D. | 2015 | Randomized clinical trial comparing neurological outcomes after carotid endarterectomy or stenting | Br J Surg | 150 |
| **C94** | Rosenfield, K. and Matsumura, J. S. and Chaturvedi, S. and Riles, T. and Ansel, G. M. and Metzger, D. C. and Wechsler, L. and Jaff, M. R. and Gray, W. and Investigators, Act I. | 2016 | Randomized Trial of Stent versus Surgery for Asymptomatic Carotid Stenosis | N Engl J Med | 1453 |
| **C95** | Cristalli, A. and Arlati, S. and Bettinelli, L. and Bracconaro, G. and Marconi, G. and Zerbi, S. | 2009 | Regional anesthesia for carotid endarterectomy: a comparison between ropivacaine and levobupivacaine | Minerva Anestesiol | 48 |
| **C96** | Walsh, S. R. and Nouraei, S. A. and Tang, T. Y. and Sadat, U. and Carpenter, R. H. and Gaunt, M. E. | 2010 | Remote ischemic preconditioning for cerebral and cardiac protection during carotid endarterectomy: results from a pilot randomized clinical trial | Vasc Endovascular Surg | 70 |
| **C97** | Umbrain, V. J. and van Gorp, V. L. and Schmedding, E. and Debing, E. E. and von Kemp, K. and van den Brande, P. M. and Camu, F. | 2004 | Ropivacaine 3.75 mg/ml, 5 mg/ml, or 7.5 mg/ml for cervical plexus block during carotid endarterectomy | Reg Anesth Pain Med | 93 |
| **C98** | Palombo, D. and Lucertini, G. and Mambrini, S. and Zettin, M. | 2007 | Subtle cerebral damage after shunting vs non shunting during carotid endarterectomy | Eur J Vasc Endovasc Surg | 96 |
| **C99** | Messner, M. and Albrecht, S. and Lang, W. and Sittl, R. and Dinkel, M. | 2007 | The superficial cervical plexus block for postoperative pain therapy in carotid artery surgery. A prospective randomised controlled trial | Eur J Vasc Endovasc Surg | 46 |
| **C100** | de Sousa, A. A. and Filho, M. A. and Faglione, W., Jr. and Carvalho, G. T. | 2005 | Superficial vs combined cervical plexus block for carotid endarterectomy: a prospective, randomized study | Surg Neurol | 125 |
| **C101** | De Castro, V. and Godet, G. and Mencia, G. and Raux, M. and Coriat, P. | 2003 | Target-controlled infusion for remifentanil in vascular patients improves hemodynamics and decreases remifentanil requirement | Anesth Analg | 46 |
| **C102** | Payne, D. A. and Jones, C. I. and Hayes, P. D. and Naylor, A. R. and Goodall, A. H. | 2007 | Therapeutic benefit of low-dose clopidogrel in patients undergoing carotid surgery is linked to variability in the platelet adenosine diphosphate response and patients' weight | Stroke | 56 |
| **C103** | Camerani, S. and Capuzzo, M. and Ieffa, E. and Pescolderung, M. and Braccini, L. and Volta, C. A. | 2014 | Total intravenous anesthesia with superficial cervical block or morphine transition in patients undergoing carotid endarterectomy | Minerva Anestesiol | 64 |
| **C104** | Kuzkov, V. V. and Obraztsov, M. Y. and Ivashchenko, O. Y. and Ivashchenko, N. Y. and Gorenkov, V. M. and Kirov, M. Y. | 2018 | Total Intravenous Versus Volatile Induction and Maintenance of Anesthesia in Elective Carotid Endarterectomy: Effects on Cerebral Oxygenation and Cognitive Functions | J Cardiothorac Vasc Anesth | 40 |
| **C105** | Borum, S. E. and Bittenbinder, T. M. and Buckley, C. J. | 2000 | Transesophageal atrial pacing reduces phenylephrine needed for blood pressure support during carotid endarterectomy | J Cardiothorac Vasc Anesth | 36 |
| **C106** | McMahon, G. S. and Jones, C. I. and Hayes, P. D. and Naylor, A. R. and Goodall, A. H. | 2013 | Transient heparin-induced platelet activation linked to generation of platelet 12-lipoxygenase. Findings from a randomised controlled trial | Thromb Haemost | 43 |
| **C107** | Hoefer, J. and Pierer, E. and Rantner, B. and Stadlbauer, K. H. and Fraedrich, G. and Fritz, J. and Kleinsasser, A. and Velik-Salchner, C. | 2015 | Ultrasound-guided regional anesthesia for carotid endarterectomy induces early hemodynamic and stress hormone changes | J Vasc Surg | 60 |
| **C108** | Markus, H. S. and McCollum, C. and Imray, C. and Goulder, M. A. and Gilbert, J. and King, A. | 2011 | The von Willebrand inhibitor ARC1779 reduces cerebral embolization after carotid endarterectomy: a randomized trial | Stroke | 36 |
| **C109** | Dahi, M. and Moshari, M. and Dabir, S. and Vosoghian, M. and Tabashi, S. and Tavakoli, N. and Madadi, F. | 2023 | Effect of vitamin c infusion on cerebral oximetry during general anesthesia for carotid endarterectomy in diabetic patients | Anesth Pain Med | 50 |
| **C110** | Reiff, T. and Eckstein, H. H. and Mansmann, U. and Jansen, O. and Fraedrich, G. and Mudra, H. and Böckler, D. and Böhm, M. and Debus, E. S. and Fiehler, J. and Mathias, K. and Ringelstein, E. B. and Schmidli, J. and Stingele, R. and Zahn, R. and Zeller, T. and Niesen, W. D. and Barlinn, K. and Binder, A. and Glahn, J. and Hacke, W. and Ringleb, P. A. and SPACE-2 Investigators | 2022 | Carotid endarterectomy or stenting or best medical treatment alone for moderate-to-severe asymptomatic carotid artery stenosis: 5-year results of a multicentre, randomised controlled trial | Lancet Neurol | 513 |
| **C111** | Opperer, M. and Kaufmann, R. and Meissnitzer, M. and Enzmann, F. K. and Dinges, C. and Hitzl, W. and Nawratil, J. and Koköfer, A. | 2022 | Depth of cervical plexus block and phrenic nerve blockade: a randomized trial. | Reg Anesth Pain Med | 45 |
| **C112** | Xu, N. and Li, L. X. and Wang, T. L. and Jiao, L. Q. and Hua, Y. and Yao, D. X. and Wu, J. and Ma, Y. H. and Tian, T. and Sun, X. L. | 2021 | Processed multiparameter electroencephalogram-guided general anesthesia management can reduce postoperative delirium following carotid endarterectomy: a randomized clinical trial | Front Neurol | 255 |
| **C113** | Youssef, F. and Jenkins, M. P. and Dawson, K. J. and Berger, L. and Myint, F. and Hamilton, G. | 2005 | The value of suction wound drain after carotid and femoral artery surgery: a randomised trial using duplex assessment of the volume of post-operative haematoma | Eur J Vasc Endovasc Surg | 70 |
